# Efficient Implementation of Approximate Fourth Order *N*-Electron Valence State Perturbation Theory

**DOI:** 10.1021/acs.jctc.4c01735

**Published:** 2025-04-04

**Authors:** Emily M. Kempfer, Kantharuban Sivalingam, Frank Neese

**Affiliations:** Max-Planck-Institut für Kohlenforschung, Mülheim an der Ruhr D-45470, Germany

## Abstract

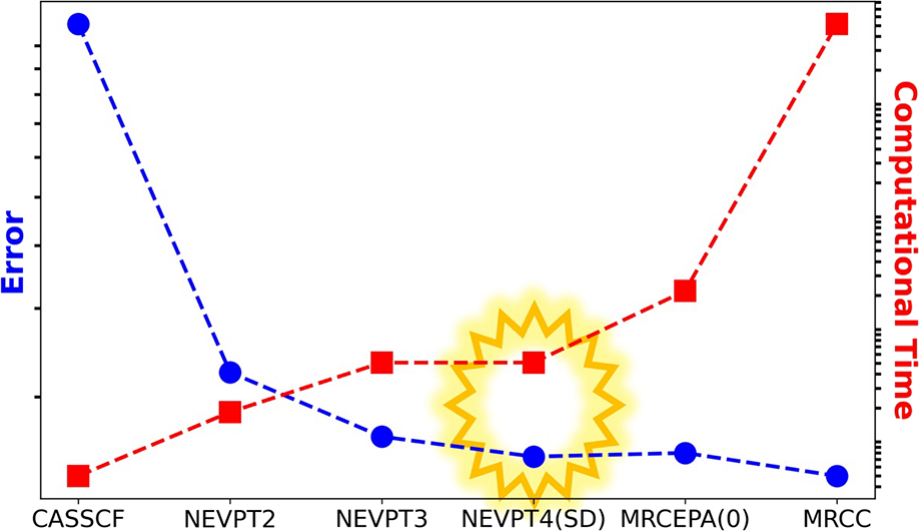

In this work, the implementation of a partial fourth order *N*-electron-valence perturbation theory (NEVPT) is reported and numerically evaluated. The method, termed NEVPT4(SD), includes the internally contracted functions that span the first-order-interacting space (FOIS) and evaluates their contribution to second-order in the wave function and fourth order in the energy. The triple- and quadruple excitations that would additionally enter the second-order-interacting space (SOIS) are not included. As discussed by Grimme [*Chem. Phys. Lett.***2001,***334,* 99–106] in order to obtain a size-consistent method, it is necessary to also drop the fourth-order renormalization term if the quadruple excitations are dropped. The NEVPT4(SD) method is demonstrated to be perfectly size consistent. Computationally, the method is still fairly affordable and requires about the same time as a single iteration of the fully internally contracted (FIC) MRCI or MRCEPA(0) and significantly cheaper than the FIC MRCC that serves as the reference for our calculations. The accuracy tests show that NEVPT4(SD) offers significant accuracy improvements over NEVPT2 for transition metal atom/ion multiplets as well as diatomic bond breaking potential energy surfaces. We find that going to fourth order in perturbation theory essentially eliminates the need for a second d-shell, thus showing that the latter primarily serves to capture higher-order dynamic correlation effects that are not present in a second-order treatment. Although it captures fourth-order correlation effects, NEVPT4(SD) is numerically not a large improvement over NEVPT2 for the calculation of Heisenberg exchange couplings as illustrated by test calculations on Cu(II) dimers.

## 1 Introduction

It has been well established in quantum chemistry that the incorporation of electron correlation via perturbation-based methodologies is able to capture electron correlation effects effectively and in a size consistent manner. While most single-reference correlated wave function calculations today are probably carried out on the basis of coupled cluster (CC) theory, many body perturbation theory (MBPT) based methods are still attractive, especially since they are of a noniterative nature and still deliver accurate results, in particular for closed-shell systems.^1^ In fact, for well-behaved systems, the results of fourth-order Möller–Plesset (MP4) perturbation theory (PT) can approach that of the gold standard coupled-cluster theory with single-, double- and perturbative triples excitations (CCSD(T)) and CCSD is often close to MP4 with single-, double- and quadruple excitations (MP4(SDQ)).^2−4^ However, there is a significant number of chemically relevant systems and electronic structure problems that will require a zeroth order wave function consisting of more than one Slater determinant (DET) or configuration state function (CSF). The addition of dynamic correlation to such a multireference (MR) zeroth order wave function is quickly becoming a formidable task. In approaching the dynamic correlation problem in the MR case, one can take two extreme points of view: (1) an uncontracted approach in which excitations are performed relative to each entity in the zeroth order reference wave function (as entity we refer here to many body expansion functions based on DETs, CSFs or spatial configurations (CFGs)),^5,6^ or (2) an internally contracted approach in which excitations are performed by acting with excitation operators onto the entire zeroth order wave function as a whole.^7,8^

The latter approach has the significant advantage that the excitation space remains roughly of the same size as in the single reference problem and the significant disadvantage that the matrix elements between such excited functions become very complex and depend on higher-order reduced density matrices over the reference wave function. In either the uncontracted (Jezorski Monkhorst Ansatz^9−11,11,11−13^) or the contracted case (full internal contraction FIC^14−18^) and its very many variants, CC theory remains highly complex. On the other hand, configuration interaction (CI) and PT approaches are relatively straightforward to generalize to the MR case. Unfortunately, MRCI suffers from well-known size-consistency problems and still high computational cost.^8,19−26^ Similarly, multi-reference coupled electron pair approximation (MRCEPA) may either be susceptible to the occurrence of intruder states or lack orbital invariance, which will compromise its reliability.^27,28^ Furthermore, methods such as multi-reference averaged quadratic coupled-cluster (MRAQCC) and multi-reference averaged coupled pair functional (MRACPF) also encounter challenges with regard to size consistency.^29−31^

For the reasons discussed above, MRPT has emerged as the most practical tool for real-life calculations including dynamic electron correlation for the MR case. In fact, here the internally contracted MRPT methods and their variants like t-NEVPT2 developed by Sokolov et al.,^32,33^ perturbation based multireference driven similarity renormalization group (MR-DSRG) proposed by Evangelista et al.,^34−38^ and the multireference adiabatic connection by Pernal et al.^39,40^ have been proven to be particularly useful. Of the very many variants that have been proposed the two methods that have found widespread application are the complete active space (CAS) self-consistent field (SCF) perturbation theory to second order (CASPT2) from Roos and co-workers^27,41^ and the *N*-electron valence perturbation theory to second order (NEVPT2) by Angeli, Malrieu, and co-workers.^42−44^ Both methods are truncated at second-order in perturbation theory. Other than technical details, the main point in which CASPT2 and NEVPT2 differ is that CASPT2 utilizes a zeroth-order Hamiltonian that is one body in nature, while the NEVPT2 method relies on the Dyall Hamiltonian^45^ that partially incorporates two body terms. The usage of the Dyall Hamiltonian for the NEVPT methodology provides two advantageous properties: size consistency and the absence of intruder states.^46^ Details of the NEVPT2 methodology can be found in refs (42–44, 47 and 48) and a short summary of the methodology will be provided in Section 2.

Second-order perturbation methods are widely recognized for their ability to adequately describe both dynamic and static correlations within a system. Demonstrating ability in accurately modeling molecular magnetism,^49,50^ describing excited states in photochemistry,^51,52^ and the understanding electronic structures of transition metal complexes.^53−55^ Spin dependent properties can conveniently be treated by quasi-degenerate perturbation theory^56,57^ or more sophisticated variants such as dynamic correlation dressed CAS^58,59^ or quasi-degenerate versions of NEVPT2.^60−62^

While the strength and weaknesses of the individual methods vary slightly with application and system, it is clear that the restriction to second-order in perturbation theory does limit the achievable accuracy somewhat. Given the limitations of second-order perturbation theory, one might consider extending the analysis to higher orders. The evidence gathered from single-reference correlation methods indicates that major improvements are obtained at fourth order^63,64^ and some evidence points to improvements at third order within multireference perturbation theory.^36,37,65−67^ However, it is well documented that third-order perturbation corrections are often counterproductive in single-reference theory and Grimme has also been skeptical about third-order corrections in his work on fourth order MRPT.^64^ Hence, the available evidence suggests that a sweet-spot in the cost to accuracy ratio may well be reached at fourth-order, which is the focus of the present work.

At fourth order in PT, the MRPT energy has contributions from the singles and doubles (*E*_SD_), the quadruples (*E*_Q_) and the triples (*E*_T_) excitations together with a renormalization term (*E*_RN_) that restores size-consistency. Grimme has analyzed an uncontracted variant of fourth order MRPT and has concluded that *E*_Q_ and *E*_RN_ nearly cancel each other.^64^ The resulting method did not contain triple or quadruple excitations. However, it did show improvements over second-order PT but was deemed too computationally expensive for real-life applications.

Thus, it appears to us, that it is important to explore the feasibility and accuracy of higher-order MRPT approaches as a promising avenue to highly accurate MR calculations on challenging systems. In this work, we will explore the fully internally contracted^68^ variant of the fourth order NEVPT variant with single-, double-excitations where quadruple and renormalization terms are excluded (NEVPT4(SD)). This method can be thought of a generalization of MP4(SDQ) to the MR case. Future work will explore the incorporation of triple and quadruple excitations. We will show below that by incorporating higher-order terms, NEVPT4(SD) is significantly more accurate than NEVPT2 and is computationally still relatively affordable since it remains no more expensive than a single iteration of the FIC-MRCEPA(0) method.

## 2 Theory

As customary in perturbation theory, the wave function and the total energy are expanded in orders of the perturbation.
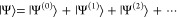
1
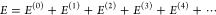
2Likewise, the Hamiltonian is divided into a zeroth order Hamiltonian,  and a perturbation *V̂*.
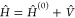
3

Using the Wigner 2*n* + 1 rule, one obtains the first-through fifth order energy as
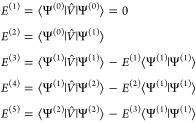
4

The second terms on the right-hand sides of *E*^(3)^, *E*^(4)^, and *E*^(5)^ are renormalization terms.
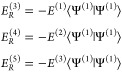
5

In the NEVPT construction utilized in this work, the first-order energy is zero, *E*^(1)^ = 0, a property specific to this approach but not generally applicable, thus the renormalization term in *E*^(3)^ is zero. At zeroth order, we have
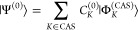
6as the CAS wave function with CI coefficients *C*_*K*_^(0)^ and CAS expansion functions |Φ_*K*_^(CAS)^⟩ that are either DETs or CSFs.

Excited CSFs are constructed by the action of spin-traced excitation operators.
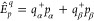
7

On the entire zeroth order wave function |Ψ^(0)^⟩ ≡ |0⟩. As usual *q*_σ_^+^ and *p*_σ_ are Fermion creation and destruction operators for an electron in orbitals *p* and *q*, respectively. The orthonormal orbital space is divided into the occupied (*i*, *j*, *k*, *l*,···), active (*t*, *u*, *v*, *w*,···), and virtual (*a*, *b*, *c*, *d*,···) subspaces. In order to simplify the notation we will not write out specific details and only mention that there are eight distinct classes of operator products , that, upon action on the zeroth order wave function, |0⟩, span the first-order interacting space (FOIS). These CSFs are, in general not orthonormal.^20,42,43^ They can, however, be brought in a form where they are orthonormal and also diagonalize the zeroth order Hamiltonian. The zeroth order Hamiltonian in the NEVPT series is the projection of the Dyall Hamiltonian in the appropriate first order interacting space.^45^ It should be noted that this process is different in the SC-NEVPT2 and FIC-NEVPT2 methodologies as noted by Angeli et al. in reference (42).

In the NEVPT series, the zeroth order Hamiltonian is taken to be the Dyall-Hamiltonian.^42,45^
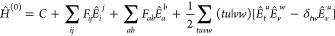
8

Here, *F*_*ij*_ is a matrix element of a suitable Fock operator, *C* is a constant that guarantees that the zeroth order energy is the CAS energy and (*tu*|*vw*) is a two-electron integral in Mulliken notation. In NEVPT, the Fock operator is state–state specific and chosen pseudocanonical, such that the inactive and virtual spaces canonicalize the Fock, whereas the active orbitals are natural. In the NEVPT methods reported here, we choose a different path and employ the pseudocanonical state-averaged orbitals for all states avoiding additional integral transformations. The resulting state-specific Fock operator is assumed to be canonical.^52^ In future publications, we will investigate this approximation in detail.

In order to simplify the notation, we will write for an excited CSF in the FOIS
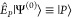
9

And assume that
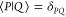
10
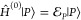
11where  is the energy of the *P*’th excited CSF.  is a simplified notation in which *P* serves as a compound label involving four actual orbital labels and all the necessary steps that lead from the elementary internally contracted CSFs to the orthonormal set that diagonalizes the zeroth order Hamiltonian have been absorbed into the definition of . For further details on the NEVPT2 methodology we refer the reader to the extensive and concise work done by Angeli et al.^42−44,48,69^

The first order wave function is expanded in terms of the FOIS
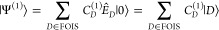
12where “*D*” is signifying double excitations. The first-order wave function is obtained from the following linear equation system.
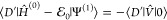
13

Noting that  is equal to the CAS energy, *E*^(0)^. This immediately leads to
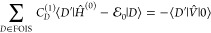
14

And given that the excited CSFs diagonalize the zeroth order Hamiltonian, we have
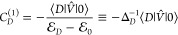
15

The second- and third-order energies are then
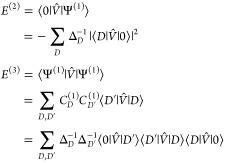
16

It is computationally advantageous to form the vector
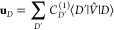
17

This vector **u** is essentially the sigma-vector in an internally contracted MRCI calculation evaluated with the first-order amplitudes.^25,68^ Consequently, the computational cost associated with evaluating it amount to the cost of one iteration in such a calculation and the third-order energy is simply
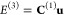
18

The second-order wave function will contain triple and quadruple excitations that span the second-order interacting space (SOIS). In order to make this construction somewhat more explicit, we will write
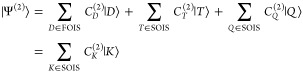
19where the second-line is a short-hand notation that includes all the SOIS CSFs. The linear equation system to be solved for the determination of the second-order wave function is
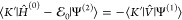
20

If we assume that the construction of the excited CSFs is carried through in a way that the CSFs of each excitation level and class are orthogonal and still diagonalize the zeroth order Hamiltonian, then we get the simple result
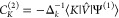
21

For the second-order doubles, this means that
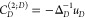
22where the notation *C*_*D*_^(2;*D*)^ implies the part of the second-order amplitude vector pertaining to the FOIS. This leads to the fourth order energy contribution from the FOIS excitations to be
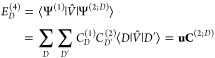
23

For the contributions of the triples and quadruples, we likewise obtain
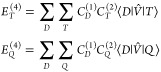
24

We are now in a position to assemble the complete fourth-order energy as
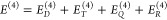
25

In his work on fourth order multireference perturbation theory, Grimme argued that the renormalization term breaks size consistency.^64^ This conclusion arises from the fact that both the norm of Ψ^(1)^ and the second-order energy scale linearly with system size. Consequently, the renormalization term scales quadratically with system size leading to the breakdown of size consistency. Furthermore, Grimme argued that this term is almost perfectly canceled by the quadruples contribution. Consequently, it is a good approximation to drop these two terms together and avoid the extremely costly calculation of the quadruples contribution altogether.^64^ In this work, we will adopt this approximation and, in addition, reserve a discussion of the triples term for a future publication. Hence, we focus on the version of NEVPT4 that amounts to the adaptation of Grimme’s fourth order MRPT to the NEVPT framework and use the acronym NEVPT4(SD), where only single and double excitations are utilized and the renormalization and quadruple excitation terms have been dropped. The version that includes the triple excitations would then be called NEVPT4(SDT).

In the NEVPT methodology, reduced order density matrices appear in the construction and diagonalization of the Dyall Hamiltonian matrix elements as well as the interaction of the excited CSFs with the perturbation operator *V̂*.^44^ Using the rank reduction scheme proposed by Dyall,^45^ at most the fourth order RDM is required for the NEVPT2, NEVPT3, and NEVPT4(SD) implementations.

## 3 Implementation

The above documented equations were implemented into a development version of the ORCA program^70^ based on ORCA 6.0. The implementation was accomplished using the ORCA-AGE toolchain^71^ that is described in full detail elsewhere. The implementation makes use of the previously developed FIC-NEVPT2 implementation inside ORCA-AGE that served as initial guess to the FIC-MRCI method described elsewhere.^68^ The computationally most expensive step is the calculation of the FIC-MRCI sigma vector with the first-order amplitudes. This step is essentially accomplished using the previously developed code. However, the big advantage of the automatic code generation chain in ORCA-AGE is its deep-integration with the ORCA code that allows for automatic regeneration of all generated code at the state-of-the-art of the existing tool chain. Hence, the present implementation of the FIC-MRCI sigma-vector is significantly more efficient than the original implementation from 2015 due to the improvements described in ref (71).

## 4 Results and Discussion

All subsequent calculations were run within the ORCA 6.0 framework. Details of basis set and computational methodology are stated within their respective sections. For further simplicity, the FIC-notation for methods has been removed from method titles. It should be noted that unless stated explicitly, all methods within the following sections are FIC-methodologies. We have also reduced the NEVPT4(SD) method notation to only NEVPT4.

### 4.1 Transition Metal Ions

In the initial benchmark set, we build on previous work^72,73^ by exploring the state-averaged excitation energies of 16 2+ and 3+ transition metal ions with the DKH-def2-QZVPP basis set. Beyond merely assessing accuracy, this study also aims at evaluating the influence of the double shell effect as well as the dependence of the results in the specific choice of orbitals.

**Figure 1:**
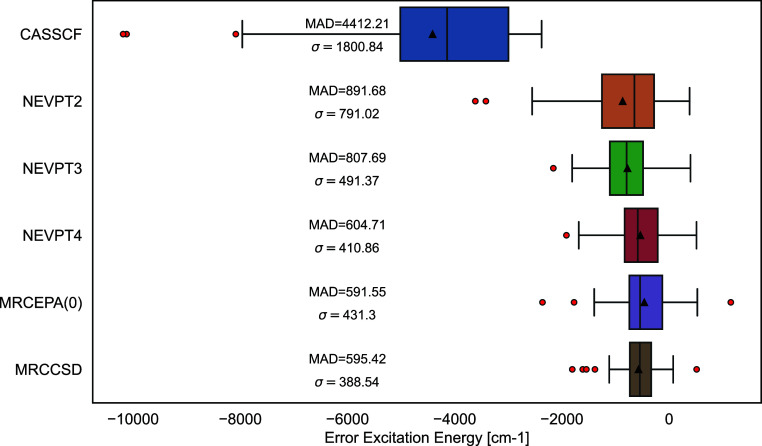
Benchmark excitation energies of 16 2+ and 3+ transition metal ions using only 3d orbital active spaces. Red circles signify outlier data points, the boxes signify where the majority of the data values fall, the line in the box is the median line, the black triangle indicates the mean value, and the whiskers are the range of the data.

**Figure 2:**
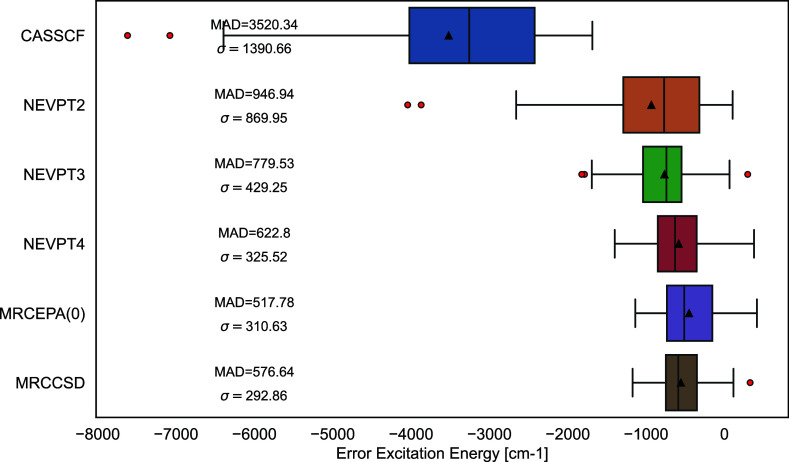
Benchmark excitation energies of 16 2+ and 3+ transition metal ions using the 3d and 4d orbital active spaces. Red circles signify outlier data points, the boxes signify where the majority of the data values fall, the line in the box is the median line, the black triangle indicates the mean value, and the whiskers are the range of the data.

The double shell effect is used frequently to incorporate some additional dynamic correlation effect into a CASSCF/CASPT2 or CASSCF/NEVPT2 study where it presumably improves the results by compensating for some shortcomings of the low order perturbation treatment of the dynamic correlation.^74−77^ Hence, it is interesting to investigate whether this, quite expensive and limiting, way of improving MRPT calculations is still necessary as one goes to higher orders in perturbation theory (Figures 1 and 2).

In this study, active spaces were systematically varied. For calculations utilizing only 3d orbitals, the active spaces included either the five metal d-orbitals (CAS(*n*,5)) or the five d-orbitals along with the 4s orbital (CAS(*n*,6)). To examine the double-shell effect, the active spaces were expanded to include the 4d orbitals, resulting in ten metal d-orbitals (CAS(*n*,10)) or ten metal d-orbitals with the 4s orbital (CAS(*n*,11)). The target states and multiplicities were averaged and compared directly to experimental data provided from the NIST tables.^78^ Experimental data uses the degeneracy-weighted multiplet average to account for the exclusion of spin–orbit coupling in our computations. Further information on this calculation is provided in the Supporting Information.

It is evident from Figure 1 that the results of state averaged CASSCF calculations are not satisfactory and deliver an error over 4400 cm^–1^ for a minimal active space. This error drops by about 20% to close to 3500 cm^–1^ upon inclusion of the second d-shell, Figure 2. Clearly, NEVPT2 is a major improvement since already for the minimal active space it drops the average absolute error to about 892 cm^–1^. Incorporating further correlation effect at the level of NEVPT3 and NEVPT4 bring further improvements to yield MAD’s of 808 and 605 cm^–1^ respectively. Interestingly, the NEVPT4, MRCEPA(0) and MRCCSD perform similarly with errors around 600 cm^–1^. It is pleasing to see that the accuracy of the NEVPT4 method is not far below the much more elaborate methods of MRCEPA(0) and MRCCSD.

Moving to the double shell results, Figure 2, it is interesting to note that the NEVPT2 and NEVPT4 results do not improve and yield an even higher error (946 and 623 cm^–1^) than with a minimal active space (892 and 605 cm^–1^) thus pointing to an error cancellation that renders the minimal active space NEVPT2 and NEVPT4 results artificially accurate. At the NEVPT3 level, the results improve from a MAD of 808 to 780 cm^–1^ upon inclusion of the second shell, which is more in line with expectations. Even with the slight increase in MAD for the NEVPT4 results, it is clear, that the need for the double shell is diminished. The double shell effect serves mainly to compensate for dynamic correlation that is missing in the subsequent perturbation calculation. As one incorporates more of the missing dynamic correlation into the treatment, the need for a second d-shell diminishes significantly. It is therefore pleasing to see that at the MRCCSD level, where the dynamic correlation effects inside the FOIS are nearly fully incorporated, there is practically no improvement to be had from introducing a second d-shell.

### 4.2 Diatomic Dissociation Potential Energy Surfaces

**Figure 3:**
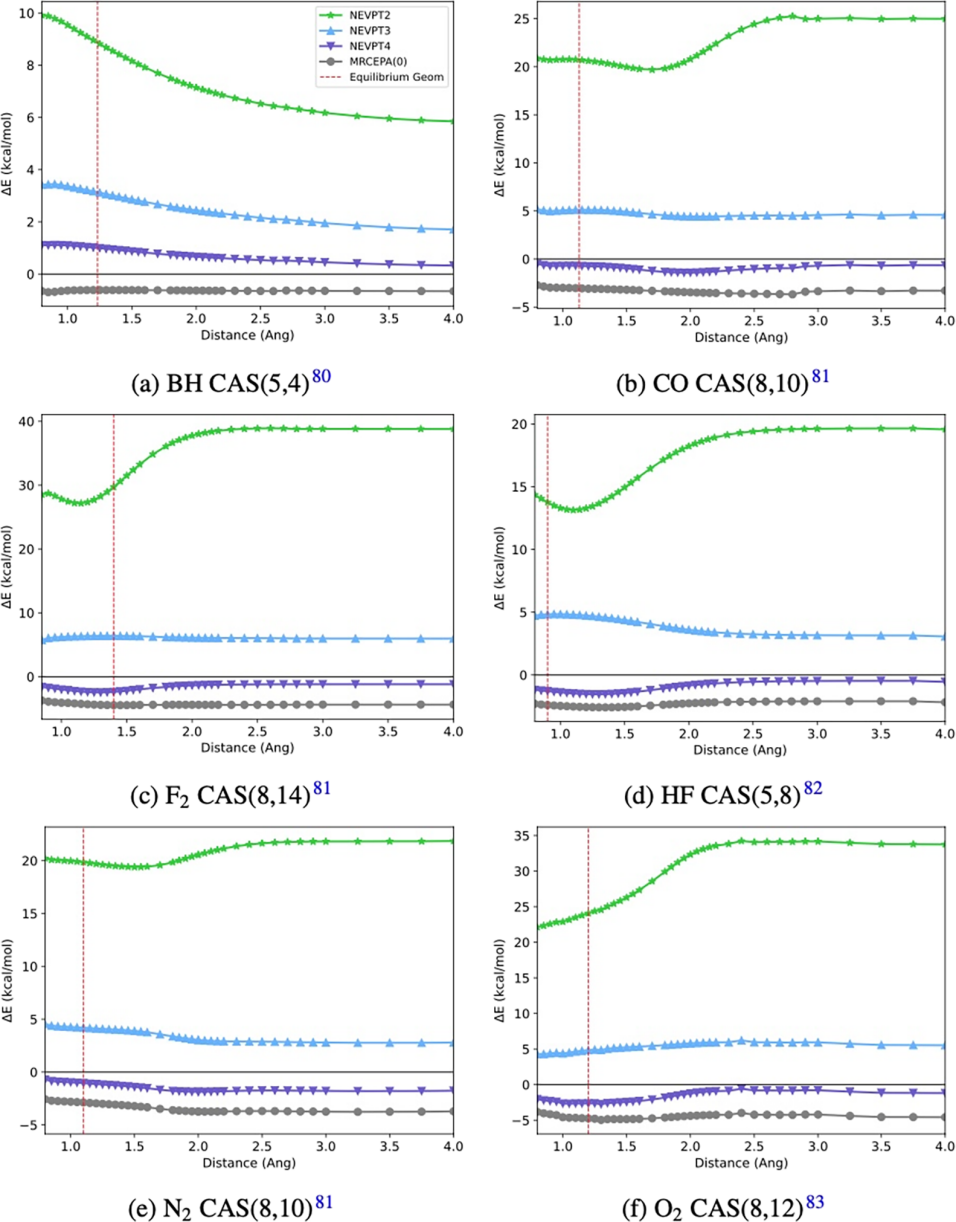
Nonparallelity errors along diatomic bond dissociation curves relative to MRCCSD at a def2-QZVP basis set with active space sizes shown in parentheses. The equilibrium geometry was maintained from the corresponding references for each diatomic noted in superscript.

One of the most stringent tests for correlation methods is to study their behavior during bond breaking along potential energy surfaces. The most important measure is the nonparallelity error relative to the reference data. In Figure 3 we examine the nonparallelity with respect to the MRCCSD reference for six diatomic PESs of BH, CO, F_2_, HF, N_2_, and O_2_. MRCCSD was the highest-level reference that was available to us. It is well-known, of course, that there is a number of closely related approaches to internally contracted MRCCSD. Except for the omission of the reference wave function relaxation, the current ORCA implementation is identical to the “ic-MRCC A” approach reported by Hanauer and Köhn,^16^ where the excitation space is spanned by the same excitation operators that are also present in the NEVPT2 methodology. In line with Hanauer and Köhn, the BCH is truncated after the quadratic commutator. In our implementation, no terms have been dropped and no approximations to the RDM’s are made.^71^ For three of the six test systems (BH, HF and N_2_), one can find high-quality FCI results in the literature.^16,79^ In an effort to ensure that the MRCCSD results provide a valid reference points we have added a comparison between FCI, MRCCSD and NEVPT4 in the Supporting Information (Section 2.2). The results show that MRCCSD is a good approximation to the FCI result, even if the nonparallelity error is not perfectly constant. In fact, the accuracy of MRCCSD and NEVPT4(SD) relative to FCI is very similar for these three systems, as can be expected from the closeness of these two methods in the test results obtained in this section. The relative active spaces were selected as the full valence space for each diatomic and the equilibrium geometries for the PESs were found within the literature.^80−83^

It can be seen from Figure 3 that the NEVPT4 methodology performs well throughout the entirety of the PES for all diatomics. For CO, F_2_, HF, N_2_, and O_2_ we can examine that the NEVPT4 methodology performs very similar to that of MRCEPA(0). However, the latter method overshoots the MRCCSD reference data significantly while NEVPT4 may be slightly below or above the reference results indicating a more balanced behavior. As expected, the NEVPT4 results nicely converge toward the reference results at long distances, while slightly larger but still highly systematic deviations exist around equilibrium distances. Nevertheless, the improvement that NEVPT4 offers over NEVPT2 and NEVPT3 are fairly striking.

The results from Figure 3 were compressed into Table 1, where, it is clearly demonstrated that NEVPT4 shows the smallest minimum and maximum value of −1.68 and −0.30 kcal/mol, respectively. It is also clear that while the MRCEPA(0) methodology may show good nonparallelity, it overestimates the energies with respect to MRCCSD. Overall, the data indicates that NEVPT4 offers very attractive accuracy at substantially lower cost than MRCCSD. Timing data will be discussed below.

**Table 1:** Maximum, Minimum, Mean Average Error (Reported in kcal/mol) and Standard Deviation of the Six Diatomic PESs with Respect to the MRCCSD Method

	NEVPT2	NEVPT3	NEVPT4	MRCEPA(0)
Max	24.99	5.11	–0.30	–2.43
Min	17.08	3.27	–1.68	–3.42
MAE	21.86	4.33	–1.00	–3.07
σ	2.78	0.52	0.43	0.24c

To ensure MRCCSD is a good reference, FCI results obtained from literature for BH,^79^ HF,^79^ and N_2_^16^ are compared to that of NEVPT2, NEVPT3, NEVPT4 and MRCCSD. The results are summarized into Table 2.

**Table 2:** Maximum, Minimum, Mean Average Error (Reported in kcal/mol) and Standard Deviation of BH, HF, and N_2_ with Respect to FCI from Literature

	NEVPT2	NEVPT3	NEVPT4	MRCCSD
Max	16.18	6.28	3.13	2.67
Min	12.29	4.07	1.15	0.96
MAE	13.85	4.80	1.85	1.52
σ	1.44	0.70	0.61	0.50

Both the NEVPT4 and MRCCSD methods tend to overestimate the FCI results. However, it is worth noting the similarity in their closeness to the FCI values. The mean absolute errors are approximately 1.85 kcal/mol for NEVPT4 and 1.52 kcal/mol for MRCCSD. While the MRCCSD method is significantly more computationally expensive, NEVPT4 achieves comparable accuracy at a much lower computational cost.

### 4.3 Ethylene Double Bond Rotation

Another important test for methodological robustness is the PES of the ethylene double bond rotation. For an accurate description of the ethylene double bond rotation, a balanced description of the π and π* orbitals are necessary and can be obtained from a CAS(2,2) calculation.^84,85^ The complexity of this system arises from the need to model the intricate interactions between these orbitals and their effects on the rotational profile. Specifically, when the molecule is within its eclipsed geometry, the singlet state is dominated by the closed shell configuration. However, increasing the rotational angle to 90° breaks the double bond leading to a dominant diradical configuration in the wave function. Within the 70° and 110° rotational conformations, the wave function undergoes its most dramatic changes and the precision of capturing these effects relies heavily on the description of dynamical correlation.^68^

**Figure 4:**
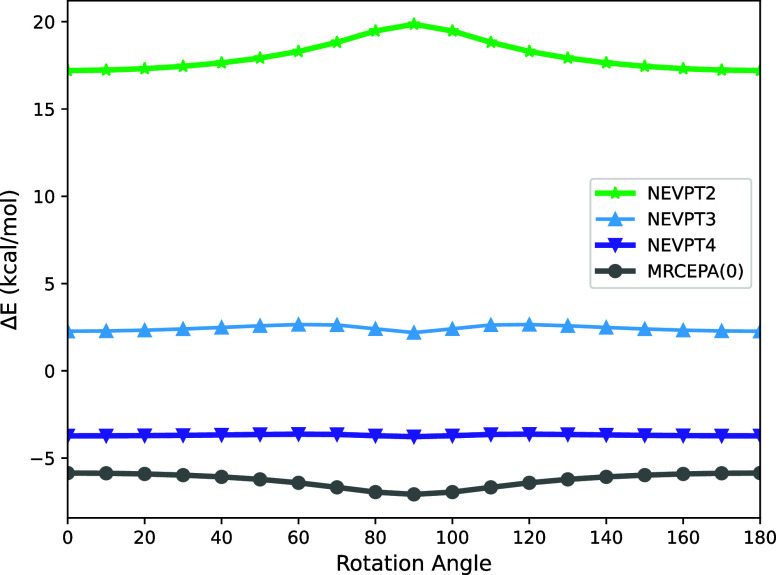
Relative error in kcal/mol of the PES of ethylene rotation of the double bond for NEVPT methodology in comparison to MRCCSD at the def2-TZVP basis set.

We present the relative errors of a geometrically rigid scanned PES of the ethylene molecule rotational profile, benchmarked against the MRCCSD/def2-TZVP method, as shown in Figure 4. The errors for each methodology range from −7 to 20 kcal/mol, with the NEVPT3 approach displaying the closest alignment to the MRCCSD reference, followed by NEVPT4. Interestingly, NEVPT3 slightly undershoots the reference values by about 2 kcal/mol while NEVPT4 overshoot it by a similar amount. However, both methods show outstanding parallelity to the reference data. In particular, for NEVPT4 there is no discernible bump close to the bond breaking point at 90°. NEVPT4 is also superior to MRCEPA(0) that overshoots the reference result by about 6 kcal/mol. MRCEPA(0) is also nicely parallel to the reference data but does show a further deterioration at 90°. These results further confirm the very attractive properties of NEVPT4 as a low-cost/high-accuracy alternative to MRCCSD.

### 4.4 Size Consistency

An important property that needs to be examined and demonstrated is the feature of size consistency. Size consistency is obtained if the energy of a noninteracting supersystem A–B, is equal to the sum of the individual systems, A and B computed individually. It is well-known that MPn methods and the NEVPT2 method are size consistent, the latter provided that the active space is chosen accordingly. And thus, we would like to provide consistent proof that increasing perturbation order in NEVPT methodologies maintains size consistent behavior. To examine this behavior, polyene dimers were examined at the NEVPT4/def2-SVP level of theory. The active spaces for the dimer systems were computed using the AVAS methodology implemented within ORCA 6.0 software to assist in the localization of the orbitals on each monomer. Active spaces and energy values are reported in the Supporting Information where it can be observed that the NEVPT4 methodology is perfectly size consistent.

### 4.5 Efficiency Test-Single Molecule with Ranging Methodologies

Before detailing the computational timings of the newly developed NEVPT4 methodology, it is essential to establish a baseline for the existing NEVPT2 methodologies. A key distinction lies between the strongly contracted NEVPT2 (SC-NEVPT2) and the fully internally contracted NEVPT2 (FIC-NEVPT2). A timing comparison of these methods was performed using decapentane CAS(10,10) with a def2-SVP basis set, Table 3. SC-NEVPT2 is known to be computationally more efficient as it is more compact and avoids the storage of the amplitudes. In contrast, the reported FIC- implementation involves amplitude storage. This computational efficiency is reflected in its shorter computational time of 72.3 s. The SC scheme works nicely and enables the application of NEVPT2 to fairly large systems. However, as shown earlier one loses unitary invariance in the active space which has negative consequences for higher-order methods such as MRCC^68,69^ or local reference correlation treatments (DLPNO-NEVPT2).^86^ Conversely, FIC-NEVPT2 employs a fully contracted approach and results in a higher computational time of 184.6 s.

**Table 3:** Computational Timings for SC-NEVPT2 and FIC-NEVPT2 for Decapentane with a (10,10) Active Space Using def2-SVP Basis Set

method	time (s)
SC-NEVPT2	72.3
FIC-NEVPT2	184.6

**Figure 5:**
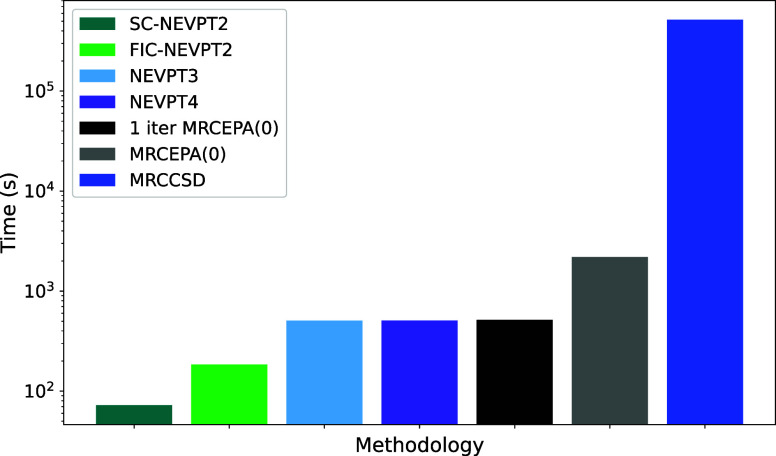
Comparison of methodological timings for the calculation of decapentane with a CAS(10,10) active space using def2-SVP basis set with SC-NEVPT2, FIC-NEVPT2, NEVPT3, NEVPT4, single iteration MRCEPA(0), MRCEPA(0), and MRCCSD methodologies in a logarithmic scale.

We can now compare the computational times of the SC- and FIC-NEVPT2 methodologies with those of NEVPT3, NEVPT4, a single iteration of MRCEPA(0), MRCEPA(0), and MRCCSD, as shown in Figure 5. The NEVPT3 method, requires 506.8 s, which is approximately 300 s longer than FIC-NEVPT2. In contrast, the NEVPT4 method, which incorporates additional complexity for improved accuracy, takes 507.8 s, making it only slightly more expensive than NEVPT3, but offering enhanced accuracy.

For methods that involve more configurations, the computational cost increases significantly. The MRCEPA(0) and MRCCSD methods take 2194.0 and 517,454.9 s, respectively. Interestingly, the computational cost of a single iteration of MRCEPA(0) is similar to that of NEVPT4, as the sigma vector is computed only once in NEVPT4, while it is recalculated with each iteration in MRCEPA(0).

**Figure 6:**
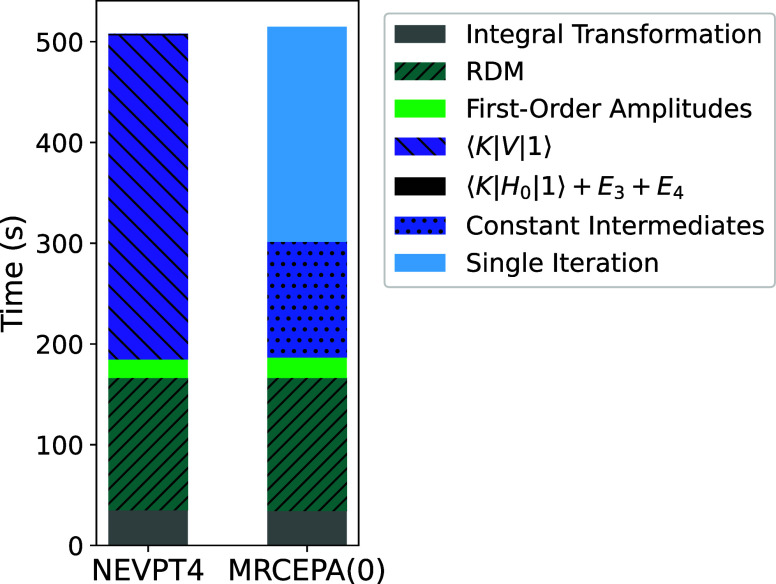
Breakdown of AUTO–CI methods of the NEVPT4 and single iteration MRCEPA(0) for decapentane with a CAS(10,10) active space using def2-SVP basis.

We can also take a deeper dive, and breakdown the timings of the NEVPT4 and single iteration MRCEPA(0) methodologies to determine where the most computational time is being spent, Figure 6. It is clearly observed that for the NEVPT4 methodology, the largest computational cost comes from the calculation of the sigma vector, or the right-hand side of the NEVPT4 calculation. This just demonstrates that the largest improvement could come from the improvement of the sigma vector calculation within the NEVPT4. Nevertheless, a time increase of only a factor of 2–3 in NEVPT4 relative to FIC-NEVPT2 is a highly encouraging result.

Other significant steps are the calculation of the higher order reduced density matrices, the initial integral transformation, as well as the determination of the first-order amplitudes. Since techniques are known to accelerate these steps, we believe that NEVPT4 could be significantly further improved.

### 4.6 Single Alkene with Increasing Active Space Size

**Figure 7:**
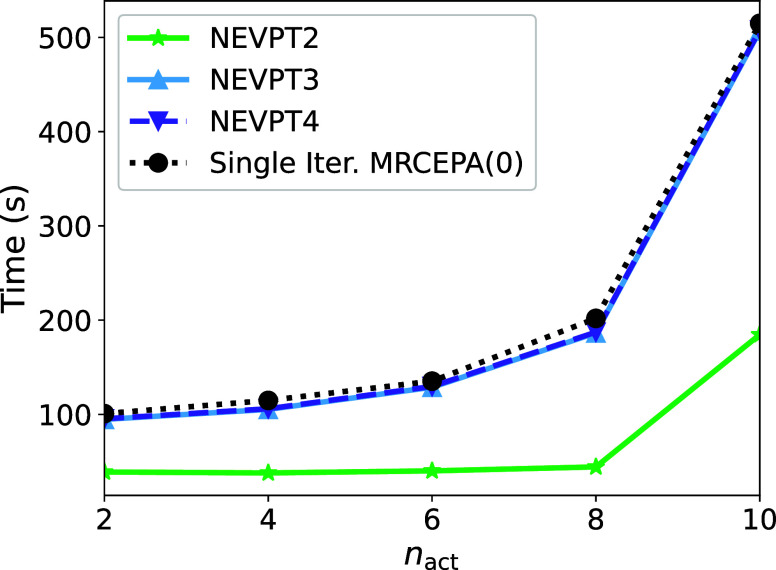
Scaling of the NEVPT4 methodology in comparison to NEVPT2, NEVPT3, and a single iteration of the MRCEPA(0) method for decapentane with ranging active space between CAS(2,2) and CAS(10,10).

We next would like to examine a single molecule with increasing active space, specifically, the decapentane molecule with active spaces ranging from CAS(2,2) to CAS(10,10) with a basis set of def2-SVP. Here, we examine the scaling of the NEVPT4 methodology with respect to NEVPT2, NEVPT3, and a single iteration of MRCEPA(0), Figure 7. It is evident that the NEVPT4 methodology exhibits the same scaling behavior as the NEVPT3 method. The similar computational cost of the NEVPT3 and NEVPT4 methods can be attributed to the calculation of the **u** vector in eq 17 that amounts to a single calculation of the closely related FIC-MRCI method which scales as  with respect to system size. The remaining steps in NEVPT3 and NEVPT4 are all computationally negligible resulting in nearly identical execution times. The scaling with respect to system size is, as expected, . A numerical example is provided in the Supporting Information (Section 7).

As the active space size increases, particularly at the CAS(10,10) active space, the computational cost of the methodology is largely dominated by the calculation of the 4-RDM. This calculation scales as , making it the primary computational bottleneck for large active spaces. This issue will be further examined in the following section.

### 4.7 Scaling with Respect to Molecular Size

**Figure 8:**
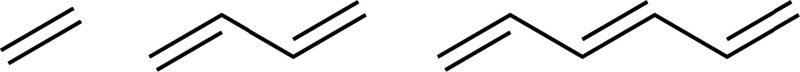
Linear, all-(*E*)-polyunsaturated alkenes from ethene to hexatriene. Each polyene can be treated with active spaces CAS(2*n*,2*n*), where *n* is the number of double bonds in the system.

We now turn to systems with large active spaces, where we can investigate efficiency of the methodology with respect to other computationally costly methodologies. Thus, we examine seven all-(*E*)-polyunsaturated alkenes (polyenes) from ethene CAS(2,2) to tetradecapentane CAS(14,14) in the def2-SVP basis set demonstrated in Figure 8.

**Figure 9:**
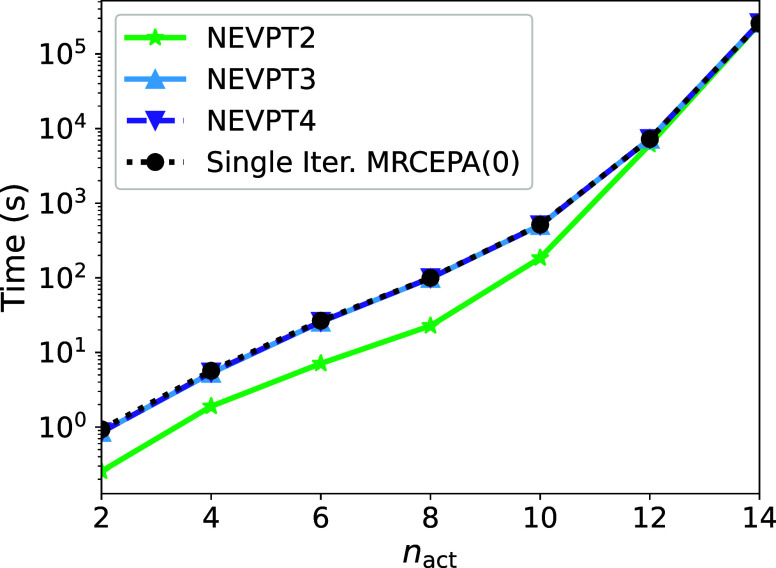
Logarithmic scaling of NEVPT4 methodology in comparison to NEVPT2, NEVPT3, and a single iteration of the MRCEPA(0) method for increasing polyene molecules with CAS(2*n*,2*n*) active spaces.

To begin evaluating the results of this study, we first focus on the computational cost of the NEVPT4 method compared to NEVPT2, NEVPT3, and a single iteration of MRCEPA(0) in Figure 9. It is evident that at smaller active spaces the cost of the NEVPT4 methodology is higher than that of the NEVPT2 however, as the active space is expanded into the larger regions, i.e. CAS(10,10), CAS(12,12), CAS(14,14), the NEVPT2 cost is similar to that of NEVPT4 due to the increased computational time of the RDM. We can also conclude that the NEVPT4 methodology takes about the same time as a single iteration of the MRCEPA(0) methodology, again due to the calculation of the **u** vector. Demonstrating the cost of the methodology does not out-weigh the accuracy achieved by increasing to fourth order.

**Figure 10:**
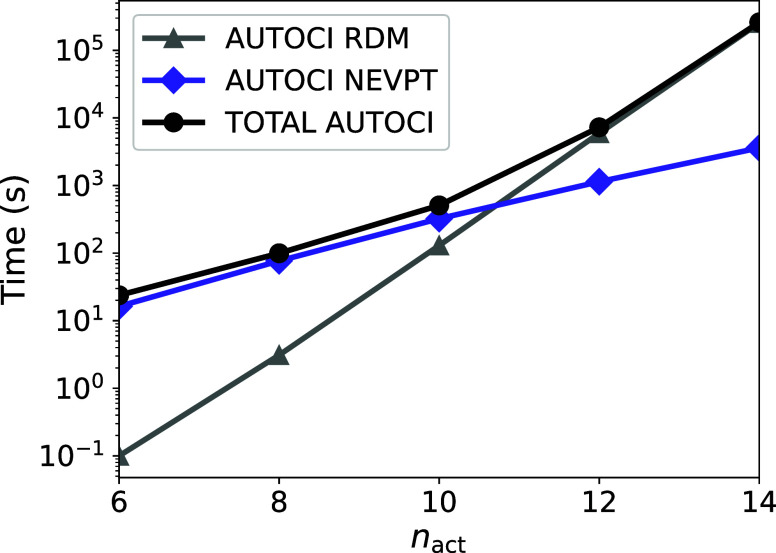
Logarithmic scaling of the total timing of the AUTOCI calculation broken into two subsequent blocks: the NEVPT calculation and the build of the RDM starting at the CAS(6,6) active space due to the negligent cost of the RDM at smaller active spaces.

We can also further break down the timing of the AUTOCI calculation. For the calculation of NEVPT4, up to the fourth order reduced density matrix (RDM) is necessary. As expected, the calculation of these matrices becomes very time-consuming as the active space increases (Figure 10). From Figure 10 it is observed that as the calculation proceeds to the larger CAS(12,12) and CAS(14,14) active spaces, the calculation of the RDM dominates the overall computation time. Thus, for such larger active spaces NEVPT4 is just as efficient as NEVPT2. Several techniques have been developed for avoiding the calculation of the 4-RDM for example cumulant decomposition,^47,87,88^ matrix product state compression,^89,90^ imaginary time propagation,^32,33^ and Monte Carlo.^91^ Since the calculation and handling of the 4-RDM quickly becomes a bottleneck for large-scale calculations given its eight power scaling with respect to the number of active orbitals, this important subject remains an active area of research.^47,92,93^

### 4.8 Antiferromagnetically Coupled Copper Complexes

One stringent test of multireference methods is the calculation of magnetic exchange interactions in spin coupled oligonuclear transition metal complexes. While the unpaired electrons on each center are usually aligned in parallel to form local high-spin states, the individual site spins can either couple ferromagnetically to a high-spin configuration or antiferromagnetically to a low-spin state. It is well-known that the energy splitting between the ferro- and antiferromagnetic states is not magnetic or relativistic in origin but simply a consequence of the Fermionic nature of electrons that requires the electronic wave function to be antisymmetric with respect to particle interchange.^41,94−98^

Evidently, even a zeroth order description of the antiferromagnetic state requires more than one Slater determinant in order for the wave function to be a spin-eigenfunction. This is automatically the case for CASSCF wave functions but can also be realized with much higher efficiency in a restricted open shell Hartree–Fock framework, as demonstrated in full generality recently.^99−103^

What is missing from such a spin-coupled SCF wave function are the ionic low-spin configurations in which unpaired electrons are being transferred from one site to another. Such configurations are present in the CASSCF wave function but their weight is dramatically underestimated due to the inflexible orbitals. Taking the simplest case of two-interacting Cu(II) ions with site-spin 1/2, it is clear that the Cu(I)/Cu(III) configurations feature rather different radial d-functions than what is optimal for the Cu(II) state. This relaxation of the orbitals can be brought about by a suitable dynamic correlation treatment that allows for “orbital breathing”. As discussed in depth by Calzado, Malrieu and co-workers, the configurations that lead to the orbital relaxation are of the one-hole/two-particle and two-hole/one-particle type whose effects should come in at fourth-order in perturbation theory.^104,105^

Given this analysis, it appears that the singlet–triplet splitting in antiferromagnetically coupled copper dimers is an ideal testing ground for the new NEVPT4 method because it nominally contains all required contributions. In this section, we will therefore report calculations for standard “guinea-pig” copper complexes ([Cu_2_Cl_6_]^2–^ and copper-acetate) together with the smallest possible and widely used H–He–H model system with the minimal active space of 2 electrons and two orbitals and basis set of def2-TZVP. Here, we will compare NEVPT2, NEVPT3, NEVPT4 to higher-level contracted or uncontracted correlation methods such as Full-CI (for H–He–H) or MRCEPA(0), Table 4.

**Table 4:** Coupling Constants Computed at CASSCF, NEVPT2, NEVPT3, and NEVPT4 Compared to Full-CI/MRCEPA(0)/Experimental Results at the def-TZVP Basis Set^e^

system	CASSCF	NEVPT2	NEVPT3	NEVPT4	ref
H–He–H	–475.0	–499.6	–504.1	–506.8	–510.3^a^/–524.1^b^
[Cu_2_Cl_6_]^2–^	11.7	14.3	26.5	28.9	–40^c^
copper acetate	–18.7	–44.9	–46.9	–62.8	–286^d^

a MRCEPA(0).

b Full-CI.

c Reference (106).

d Reference (107).

e All values are shown in cm^–1^.

In the case of the H–He–H model system, we observe a small increase in the coupling constant from NEVPT2 to NEVPT4, with the value of −506.8 cm^–1^ being close to the reference Full-CI value. The improvements observed by going from NEVPT2 to NEVPT4 are, however, limited.

For the [Cu_2_Cl_6_]^2–^ complex, the NEVPT4 yields a coupling constant of 28.9 cm^–1^, which is slightly more ferromagnetic than the CASSCF and NEVPT2 results, 11.7 and 14.3 cm^–1^, respectively. However, the experimental results show an opposite signed value of −40 cm^–1^.

The situation is further complicated for copper acetate, where NEVPT4 returns a coupling constant of −62.8 cm^–1^. This is a small improvement over the NEVPT2 and NEVPT3 values of −45 and −47 cm^–1^ but is missing the mark significantly with respect to the experimental value of −286 cm^–1^ reported in the literature.^107^

Thus, while NEVPT4 formally contains the necessary terms for the successful calculation of the Heisenberg exchange coupling, it appears that numerical evidence points toward a fourth order treatment by itself being not sufficient to reach high accuracy. The failure of NEVPT4 to reach quantitative accuracy might have a variety of reasons among them might be (1) the contracted nature of the perturbation treatment, (2) the missing triple- and quadruple-excitations and/or (3) a lack of reference wave function relaxation. We are currently unable to pinpoint the exact nature of the problem and will reserve such studies for future work. We will, however, remark that the higher excitations are unlikely to account for the discrepancies since DDCI calculations reach high accuracy and they do not contain any higher excitations either.

The copper-containing systems studied here have been the subject of extensive research due to the inherent challenges in predicting their magnetic exchange coupling constants with one challenge being the balance between the ionic and neutral contributions to the wave function. The balance between these contributions is critical for accurately describing magnetic exchange interactions, specifically when describing the singlet state of the dicopper complex.^108−110^ This balance is strongly influenced by single excitations, which allow mixing between neutral (localized) and ionic (delocalized) configurations. Methods like DDCI explicitly incorporate these single excitations, enabling a variational adjustment of the wave function in an uncontracted fashion. The accuracy of this approach has been amply documented, for example in application to [Cu_2_Cl_6_]^2–^ complex and copper acetate.^104^ The fact that DDCI is uncontracted and variational is in sharp contrast to all internally contracted multireference-perturbation approaches that all lack the ability to properly relax the reference wave function in the dynamic correlation field. Thus, while these effects reportedly enter in fourth order in perturbation theory, presumably the internal contraction prevents them from being accurately represented in NEVPT4 or similarly constructed methods.

### 4.9 Ionic V State of Ethylene

Ethylene’s V state, corresponding to the π → π* excitation, poses significant challenges to multireference methods due to its complex electronic structure. Angeli et al. and others have emphasized the difficulty of accurately describing this state, highlighting the need to incorporate single excitations to properly account for the interaction between the σ skeleton and the π orbitals.^111,112^ The ionic character of the V state is particularly relevant in this context, since the promotion of an electron from the bonding π orbital to the antibonding π* orbital leads to a charge redistribution within the molecule. This redistribution results in a state that is not purely neutral, affecting excitation energy and response to correlation effects. The V state requires a more diffuse description compared to typical valence excitations, and multireference methods tend to overestimate excitation energies if these effects are not adequately captured.^113^

The ionicity of the V state wave function arises from the interaction between the π → π* excitation and the underlying σ framework, which introduces charge-transfer-like effects. Single excitations play a crucial role in describing this interaction, as they allow repolarization of the ionic and neutral components within the state. In the absence of sufficient dynamical correlation, the ionic component may be underestimated, leading to artificially high excitation energies. The ethylene molecule thus serves as an excellent model system for the evaluation of the effectiveness of the NEVPT4 methodology. The NEVPT4 methodology incorporates single excitations within the second order wave function and the fourth order energy thus, it provides a more balanced description of the V state compared to lower order methods.

An investigation was conducted on ethylene employing three different basis sets as previously performed by Angeli.^111^ The contraction schemes of the utilized basis sets are shown in Table 5. The ANO-1 basis set is a contraction mixed of both the Roos double-Zeta and triple-Zeta basis sets.^114^ The following TZ and QZ basis sets are modified aug-cc methodologies in which additional diffuse functions are added to the carbons as indicated by Müller et al.^112^

**Table 5:** Basis Set Contractions Utilized for the Examination of the Ethylene V State

basis set	carbon	hydrogen	diffuse
ANO-1	(14s9p4d3f)/[5s4p2d1f]	(8s4p3d)/[3s2p1d]	
TZ	aug-cc-pVTZ	aug-cc-pVTZ	*p* 0.011900
			*p* 0.003996
QZ	aug-cc-pVQZ	aug-cc-pVQZ	*p* 0.010727
			*p* 0.003576

The vertical excitation energies from the ground *A*_*g*_ state to the excited *B*_1*u*_ state are calculated at the ground state minimum geometry of *R*_CH_ = 1.086 Å, *R*_CC_ = 1.339 Å, and ∠HCH = 117.6°. State-averaged calculations were executed over 2 roots, *A*_*g*_ and *B*_1*u*_. Two active spaces were used, both a minimal active space of 2 electrons and 2 orbitals but also extending the active space to 6 electrons in 6 orbitals. The CAS(6,6) active space consists of the 1b_2g_, 1b_3u_, 3a_g_1b_3g_, 2b_2u_, and 3b_1u_ orbitals. The active space was examined because it includes the most important σπ excitations for the V state as discussed by Müller et al.^112^

Determining the reference energy for excitations such as the V state of ethylene is not straightforward. This is because the energy value obtained from absorption spectra cannot be used directly as a reference. The transition associated with the V state of ethylene is significantly nonvertical and involves considerable anharmonicity, making the absorption spectrum value unsuitable for comparison with computational calculations. In particular, the absorption spectrum value of 7.66 eV cannot serve as a reliable reference.^115,116^ Researchers have extensively investigated which energy value best describes the system, and we adopt the average transition energy of 7.86–7.88 eV as determined by Gatti et al.^117^

The results for the CAS(2,2) active space are summarized in Table 6. Compared to the reference value of 7.86–7.88 eV, both NEVPT2 and NEVPT4 overestimate the excitation energy. However, NEVPT4 shows a significant improvement over NEVPT2, reducing the excitation energy difference to 0.10–0.15 eV depending on the basis set. This improvement is consistent with the fact that NEVPT4 incorporates additional electron correlation effects.

**Table 6:** Excitation Energies in eV for the First *B*_1*u*_ Excited State in Reference to the Ground *A*_*g*_ State Using a CAS(2,2) Active Space at Different Basis Sets for the CASSCF, NEVPT2, and NEVPT4 Methodologies

basis set	CASSCF	NEVPT2	NEVPT4
ANO-1	8.10	8.28	7.99
TZ	7.43	8.94	8.14
QZ	7.44	9.00	8.15

The CAS(6,6) active space, which contains the σπ orbitals, was also examined. The results are shown in Table 7. As expected, the inclusion of additional orbitals leads to a more accurate description of the electronic structure. With the extended active space, NEVPT4 gives the closest agreement with the reference value, with errors of 0.07 eV.

**Table 7:** Excitation Energies in eV for the First *B*_1*u*_ Excited State in Reference to the Ground *A*_*g*_ State Using a CAS(6,6) Active Space at Different Basis Sets for the CASSCF, NEVPT2, and NEVPT4 Methodologies

basis set	CASSCF	NEVPT2	NEVPT4
ANO-1	8.31	7.99	7.93
TZ	8.22	7.96	7.87
QZ	8.22	7.98	7.87

Comparing the results of the CAS(2,2) and CAS(6,6) active spaces for the NEVPT4 method, we observe that the excitation energy decreases as the active space expands. This trend is consistent with the inclusion of additional correlation effects from the σπ orbitals, which better capture the electronic excitations in the system.

## 5 Conclusions

In this work, the implementation and evaluation of a fully internally contracted fourth-order perturbation theory, NEVPT4, is reported. In this method, the extremely costly and highly complex calculation of triple- and quadruple-excitations is avoided altogether, and only the contributions of the first-order-interacting space are taken to fourth order. As previously discussed by Grimme,^64^ this necessitates dropping the renormalization term that occurs in fourth-order perturbation theory because it interferes with size consistency and would be canceled by the quadruple excitation contributions in a full fourth-order treatment. The acronym “NEVPT4(SD)” is utilized but it is emphasized that the method includes single- and double-excitations while excluding the renormalization and quadruple-excitation terms.

Our numerical evaluation shows that NEVPT4(SD) is perfectly size consistent. A detailed comparison against internally contracted MRCCSD results demonstrates that the method offers significant improvements over NEVPT2. It is encouraging that these improvements come at a moderate computational cost. The NEVPT4(SD) method comes essentially at the cost of one iteration in the MRCEPA(0) method. Hence, NEVPT4(SD) occupies a very favorable niche in the arsenal of internally contracted multireference methods. It improves significantly over NEVPT2 and reaches, or almost reaches, the accuracy of MRCEPA(0) or MRCCSD while being about an order of magnitude faster.

While the improvements in the results for NEVPT4(SD) over NEVPT2 are very clear for transition metal multiplets and bond-breaking potential energy surfaces, the results for Heisenberg exchange couplings or ionic excitation energies have not been satisfactory. Based on the analysis of Calzado and Malrieu^104,105^ and Angeli,^111^ it was expected that the necessary physics to properly describe exchange couplings and ionic excitations would appear at fourth order in perturbation theory, even when the excitation space is restricted to the FOIS. However, unfortunately, the numerical evaluation shows that NEVPT4(SD) is barely an improvement over NEVPT2. Hence, this specific area will require further investigation.

Taken together, we believe that the NEVPT4(SD) method is a very attractive addition to the arsenal of multireference methods. Given its internally contracted construction, the representation of the excitation space remains highly compact, while the computational effort remains very reasonable and will allow the application of the method to systems with around 1000 basis functions on present-day hardware. We will report further studies and enhancements of the method in real-life applications in due course.

## Supporting Information Available

Difference excitation energies (cm^–1^) for 2+ and 3+ transition metal ions (3d and 3d + 4d active space) using CASSCF, NEVPT2, NEVPT3, NEVPT4, MRCEPA(0), and MRCCSD. Difference energies (kcal/mol) vs MRCCSD for NEVPT2, NEVPT3, NEVPT4, and MRCEPA(0) for diatomic potential energy surfaces. Difference energies (kcal/mol) vs MRCEPA(0) for NEVPT2, NEVPT3, and NEVPT4 for ethylene double-bond rotation; includes example ORCA calculation for NEVPT4. Size consistency demonstration with homodimers and heterodimers (def2-SVP basis); includes Cartesian coordinates and example ORCA calculation with AVAS. Timing records (seconds) for Sections 4.5–4.7. Energies (Hartree) for singlet and triplet states of antiferromagnetically coupled copper complexes using CASSCF, NEVPT2, NEVPT3, and NEVPT4; includes example ORCA calculation (PDF)
