# Supplementary material for: Efficient Implementation of Approximate Fourth Order N-Electron Valence State Perturbation Theory
Source: J Chem Theory Comput. 2025 Apr 4;21(8):3953–67. doi: 10.1021/acs.jctc.4c01735 (PMC12020360; doi:10.1021/acs.jctc.4c01735)
Supplement: Supplementary file 1 — ct4c01735_si_001.pdf [file ct4c01735_si_001.pdf]

Supplementary Information: Efficient Implementation of Approximate Fourth Order N-Electron Valence State Perturbation Theory

Emily M. Kempfer, Kantharuban Sivalingam, and Frank Neese*

Max-Planck-Institut für Kohlenforschung, Mülheim an der Ruhr, D-45470, Germany

E-mail: Neese@kofo.mpg.de

Contents

List of Tables **S-3**

1 Transition Metal Ions **S-6**

1.1 NIST Reference Value Calculation S-6

1.2 Difference Excitation Energies S-7

2 Diatomic Dissociation Potential Energy Surfaces **S-11**

2.1 MRCCSD comparison S-11

2.2 FCI comparison S-17

3 Ethylene Double Bond Rotation **S-21**

3.1 Example NEVPT4 ORCA Calculation S-23

3.2 Example FIC-MRCCSD ORCA Calculation S-23

4 Size Consistency **S-24**

4.1 Example ORCA Calculation S-28

5 Efficiency Test- Single Molecule with Ranging Methodologies S-29

5.1 Timings . . . S-29

5.2 AUTOCI Breakdown . . . S-29

6 Single Alkene with ranging Active space S-29

6.1 Timings . . . S-29

6.2 Example ORCA Calculation . . . S-30

7 Increasing Molecular Size S-31

8 Scaling with respect to molecular size S-32

8.1 Timings . . . S-32

9 Antiferromagnetically Coupled Copper Complexes S-33

9.1 Example ORCA Calculation . . . S-34

List of Tables

S1 Difference excitation energies in cm^{-1} with respect to experimental values obtained from NIST of various states of 3d (II) metal ion relative to the respective ground state at various multireference methodologies utilizing only 3d orbitals in the active space in a basis of DKH-def2-QZVPP. S-7

S2 Difference excitation energies in cm^{-1} with respect to experimental values obtained from NIST for various states of 3d (III) metal ion relative to the respective ground state at various multireference methodologies utilizing only 3d orbitals in the active space in a basis of DKH-def2-QZVPP. S-8

S3 Difference excitation energies in cm^{-1} with respect to experimental values obtained from NIST for 3d (II) metal ion relative to the respective ground state at various multireference methodologies utilizing 3d and 4d orbitals in the active space in a basis of DKH-def2-QZVPP. S-9

S4 Difference excitation energies in cm^{-1} with respect to experimental values obtained from NIST for 3d (III) metal ion relative to the respective ground state at various multireference methodologies utilizing 3d and 4d orbitals in the active space in a basis of DKH-def2-QZVPP. S-10

S5 Difference energy in kcal/mol in comparison to MRCCSD for NEVPT methods and MRCEPA(0) for the BH molecule in the def2-QZVP basis set. S-11

S6 Difference energy in kcal/mol in comparison to MRCCSD for NEVPT methods and MRCEPA(0) for the CO molecule in the def2-QZVP basis set. S-12

S7 Difference energy in kcal/mol in comparison to MRCCSD for NEVPT methods and MRCEPA(0) for the F_2 molecule in the def2-QZVP basis set. S-13

S8 Difference energy in kcal/mol in comparison to MRCCSD for NEVPT methods and MRCEPA(0) for the HF molecule in the def2-QZVP basis set. S-14

S9 Difference energy in kcal/mol in comparison to MRCCSD for NEVPT methods and MRCEPA(0) for the N_2 molecule in the def2-QZVP basis set. S-15

S10 Difference energy in kcal/mol in comparison to MRCCSD for NEVPT methods and MRCEPA(0) for the O_2 molecule in the def2-QZVP basis set. S-16

S11 Difference energy values (reported in kcal/mol) between FCI and NEVPT2, NEVPT3, NEVPT4, and MRCCSD inlcuding maximum, minimum, mean average error (reported in kcal/mol) and standard deviation for the BH molecule using the aug-cc-pVQZ basis set. FCI results were taken from Dutta, A.; Sherrill, C. D. *J. Chem. Phys.*,**2003**,118, 1610-1619. S-18

S12 Difference energy values (reported in kcal/mol) between FCI and NEVPT2, NEVPT3, NEVPT4, and MRCCSD inlcuding maximum, minimum, mean average error (reported in kcal/mol) and standard deviation for the N_2 molecule using the cc-pVDZ basis set. FCI results were taken from Hanauer, M.; Köhn, A. *J. Chem. Phys.*,**2011**,134. . . . . S-19

S13 Difference energy values (reported in kcal/mol) between FCI and NEVPT2, NEVPT3, NEVPT4, and MRCCSD inlcuding maximum, minimum, mean average error (reported in kcal/mol) and standard deviation for the HF molecule using the 6-31G** basis set. FCI results were taken from Dutta, A.; Sherrill, C. D. *J. Chem. Phys.*,**2003**,118, 1610-1619. . . . . S-20

S14 MRCCSD energy values in Hartree for the rotation of the ethylene double bond with a basis of def2-TZVP. S-21

S15 Difference energy values in kcal/mol in comparison to MRCCSD for NEVPT method for the rotation of the ethylene double bond with a basis of def2-TZVP. S-22

S16 Monomer and dimer absolute energies in hartree with a basis set of def2-SVP. . S-24

S17 Cartesian coordinates for ethylene dimer. S-24

S18 Cartesian coordinates for butadiene dimer. S-25

S19 Cartesian coordinates for hexatriene dimer. S-26

S20 Cartesian coordinates for ethylene+butadiene dimer. S-26

S21 Cartesian coordinates for ethylene+hexatriene dimer. S-27

S22 Timing in seconds for SC- and FIC-NEVPT2 in comparison to NEVPT3, NEVPT4, single iteration MRCEPA(0), MRCEPA(0), and MRCCSD with decapentane(10,10) in a basis set of def2-SVP. S-29

S23 Breaddown of AUTOCI Timings in seconds for NEVPT4 and a single iteration MRCEPA(0) for decapentane(10,10) in a basis set of def2-SVP. S-29
S24 Timings in seconds for NEVPT methodologies in comparison to a single iteration of MRCEPA(0) for the decapentane molecule with ranging active space with a basis set of def2-SVP. S-29
S25 Timings in seconds for NEVPT methodologies in comparison to a single iteration of MRCEPA(0) for the polyene benchmarking set with a basis set of def2-SVP. S-32
S26 Timing in seconds of the AUTOCI calculation broken into the NEVPT and RDM parts with the def2-SVP basis set. S-32
S27 Energies in Hartree for singlet and triplet states for the H-He-H molecule using the NEVPT methodologies with the def2-TZVP basis set. S-33
S28 Energies in Hartree for singlet and triplet states for $[Cu_2Cl_6]^{-2}$ using the NEVPT methodologies with the def2-TZVP basis set. S-33
S29 Energies in Hartree for singlet and triplet states for $Cu_2(\mu\text{-}CH_3COO)_4(H_2O)_2$ using the NEVPT methodologies with the def2-TZVP basis set. S-33

For further simplicity, the FIC- notation for methods has been removed from method titles. It should be noted that unless stated explicitly, all methods within the following sections are FIC-methodologies. We have also reduced the NEVPT4(SD) method notation to only NEVPT4.

1 Transition Metal Ions

1.1 NIST Reference Value Calculation

Due to the lack of spin-orbit coupling in the calculations in this study, a direct comparison to the NIST Atomic Spectra Database requires the use of degeneracy-weighted multiplet averages to remove spin-orbit effects. The "experimental" values utilized throughout this work are referred to as degeneracy-weighted multiplet averages utilizing:

$$\bar{E}(L,S) = \frac{\sum_J (2J+1)E(J,L,S)}{\sum_J (2J+1)} \tag{1}$$

In this equation, the symbol $\bar{E}(L,S)$ denotes the averaged energy of a given L,S term, while $E(J,L,S)$ signifies the energy of the individual angular momentum component for each multiplet.

1.2 Difference Excitation Energies

Table S1: Difference excitation energies in cm^{-1} with respect to experimental values obtained from NIST of various states of 3d (II) metal ion relative to the respective ground state at various multireference methodologies utilizing only 3d orbitals in the active space in a basis of DKH-def2-QZVPP.

Ion	State	Exp.	CASSCF	NEVPT2	NEVPT3	NEVPT4	MRCEPA(0)	MRCCSD
Ti(II)	$^1D(^3F)$	8231.7	-2732.7	-664.5	-283.9	-74.9	76.2	-184.9
	$^3P(^3F)$	10419.9	-2844.4	-501.6	105.1	245.0	245.6	72.2
	$^1G(^3F)$	14155.8	-2998.6	-362.6	-485.3	-216.4	-174.3	-338.4
	$^1S(^3F)$	32233.7	-10120.9	-3418.5	-1400.3	-126.9	1150.7	-323.9
V(II)	$^4P(^4F)$	11330.5	-3251.5	-515.3	31.2	161.0	151.7	-26.9
	$^2G(^4F)$	11751.7	-2934.4	-510.5	-653.0	-481.8	-447.1	-552.4
	$^2P(^4F)$	15222.9	-4323.9	-1300.4	-1394.9	-1122.1	-1088.1	-1384.1
	$^2H(^4F)$	16564.6	-2982.2	-142.3	-581.0	-391.4	-330.1	-398.4
	$^2D(^4F)$	16008.0	-5238.0	-1341.2	-600.8	-287.2	-127.2	-411.0
Cr(II)	$^3H(^5D)$	17064.7	-2722.9	-187.8	-1030.6	-828.7	-758.1	-628.0
	$^3P(^5D)$	17152.3	-4918.9	-1634.7	-1005.5	-673.4	-441.4	-414.0
	$^3F(^5D)$	18176.7	-4574.4	-1102.3	-1014.3	-749.7	-597.8	-539.9
	$^3G(^5D)$	20521.2	-4526.4	-492.3	-853.4	-630.0	-535.3	-528.2
Mn(II)	$^4G(^6S)$	26845.3	-5338.6	-664.9	-1221.9	-920.9	-792.2	-964.2
	$^4P(^6S)$	29193.2	-7962.3	-2369.9	-1553.2	-1008.0	-644.4	-962.8
	$^4D(^6S)$	32351.9	-7734.0	-1080.2	-1057.0	-727.0	-548.3	-885.0
Fe(II)	$^3H(^5D)$	19828.9	-2513.8	-33.8	-997.0	-898.0	-978.3	-832.8
	$^3P(^5D)$	19609.8	-5305.5	-1471.5	-877.8	-630.4	-576.3	-512.1
	$^3F(^5D)$	21212.4	-4476.3	-958.4	-963.8	-774.4	-772.6	-709.1
	$^3G(^5D)$	24414.4	-3876.7	-184.3	-812.1	-681.5	-725.4	-657.6
Co(II)	$^4P(^4F)$	14561.4	-4240.0	-571.0	-12.9	179.6	232.3	-161.5
	$^2G(^4F)$	16512.2	-2378.7	-301.9	-631.6	-511.0	-602.7	-593.3
	$^2P(^4F)$	19620.5	-5537.5	-1591.3	-2160.1	-1562.2	-1773.2	-1611.7
	$^2H(^4F)$	22228.0	-2930.0	312.1	-141.8	-124.2	-192.8	-352.1
	$^2D(^4F)$	22713.9	-4622.0	-970.0	-501.1	-230.3	-186.7	-303.5
Ni(II)	$^1D(^3F)$	13037.6	-3367.1	-848.1	-659.3	-489.3	-491.9	-575.2
	$^3P(^3F)$	15836.3	-4043.4	-214.0	198.1	352.8	395.9	75.8
	$^1G(^3F)$	22114.8	-3567.2	-99.9	-567.9	-412.4	-480.8	-630.5

Table S2: Difference excitation energies in cm^{-1} with respect to experimental values obtained from NIST for various states of 3d (III) metal ion relative to the respective ground state at various multireference methodologies utilizing only 3d orbitals in the active space in a basis of DKH-def2-QZVPP.

Ion	State	Exp.	CASSCF	NEVPT2	NEVPT3	NEVPT4	MRCEPA(0)	MRCCSD
V(III)	$^1D(^3F)$	10536.0	-2992.6	-793.5	-365.4	-175.3	-124.0	-290.6
	$^3P(^3F)$	12924.6	-3322.0	-631.6	80.0	222.4	200.6	28.6
	$^1G(^3F)$	17967.9	-3142.5	-363.7	-536.0	-295.0	-312.6	-453.5
	$^1S(^3F)$	42038.8	-10185.3	-3615.3	-1480.6	-322.7	395.6	-518.9
Cr(III)	$^4P(^4F)$	13757.7	-3677.4	-588.2	29.8	162.2	129.2	-23.3
	$^2G(^4F)$	14699.9	-3001.6	-500.0	-695.5	-557.7	-561.7	-615.6
	$^2P(^4F)$	18919.0	-4594.1	-1609.9	-1623.3	-1685.7	-1397.0	-1543.7
	$^2D(^4F)$	20112.2	-5473.7	-1333.1	-536.8	-245.2	-149.9	-354.3
	$^2H(^4F)$	20658.0	-2855.1	-2.0	-582.6	-360.4	-440.8	-445.1
Mn(III)	$^3P(^5D)$	21247.7	-4941.5	-1777.7	-1105.0	-794.3	-606.8	-513.7
	$^3H(^5D)$	20959.7	-2490.6	-190.9	-1143.9	-1010.3	-994.8	-803.6
	$^3F(^5D)$	22344.1	-4619.7	-1236.6	-1143.8	-913.4	-808.8	-700.3
	$^3G(^5D)$	25152.6	-4481.4	-516.4	-912.8	-749.2	-717.0	-633.2
Fe(III)	$^4G(^1S)$	32281.3	-5254.5	-683.5	-1308.9	-1073.4	-1016.7	-1083.1
	$^4P(^1S)$	35305.8	-8083.3	-2556.1	-1700.8	-1198.9	-931.8	-1117.7
	$^4D(^1S)$	38869.6	-7827.3	-1131.4	-1111.3	-837.2	-737.0	-932.6
Co(III)	$^3P(^5D)$	23166.7	-5706.2	-1661.7	-961.7	-696.6	-594.3	-575.2
	$^3H(^5D)$	23341.5	-2522.0	-120.3	-1129.2	-1051.6	-1101.1	-982.3
	$^3F(^5D)$	25028.7	-4709.4	-1132.5	-1093.6	-903.9	-861.6	-817.1
	$^3G(^5D)$	28814.2	-3886.9	-219.6	-847.8	-729.6	-736.0	-679.8
Ni(III)	$^4P(^4F)$	17189.3	-4249.3	-593.2	-77.1	69.0	90.2	-147.7
	$^2G(^4F)$	19174.5	-2502.6	-397.9	-728.2	-612.5	-644.6	-659.9
	$^2P(^4F)$	22831.0	-5992.2	-1959.0	-1809.2	-1915.8	-2362.6	-1806.8
	$^2H(^4F)$	25964.6	-2858.6	382.1	-295.1	-112.4	-66.0	-308.8
	$^2D(^4F)$	26616.9	-4729.2	-1011.5	-477.4	-200.9	-105.8	-215.8
Cu(III)	$^1D(^3F)$	14894.8	-3666.3	-1011.8	-775.0	-614.9	-588.4	-552.8
	$^3P(^3F)$	18557.4	-3816.6	-17.7	398.4	510.8	526.7	513.2
	$^1G(^3F)$	25559.8	-3439.5	-51.4	-486.7	-351.9	-407.6	-396.9

Table S3: Difference excitation energies in cm^{-1} with respect to experimental values obtained from NIST for 3d (II) metal ion relative to the respective ground state at various multireference methodologies utilizing 3d and 4d orbitals in the active space in a basis of DKH-def2-QZVPP.

Ion	State	Exp.	CASSCF	NEVPT2	NEVPT3	NEVPT4	MRCEPA(0)	MRCCSD
Ti(II)	$^1D(^3F)$	8231.7	-2272.2	-831.7	-330.8	-167.8	42.4	-290.2
	$^3P(^3F)$	10419.9	-2375.2	-743.0	33.1	160.4	250.9	115.0
	$^1G(^3F)$	14155.8	-2230.3	-326.7	-440.1	-293.6	-245.7	-411.8
	$^1S(^3F)$	32233.7	-7078.3	-3870.9	-1692.6	-776.2	366.7	-521.1
V(II)	$^4P(^4F)$	11330.5	-2758.5	-727.4	-23.7	96.7	173.4	18.0
	$^2G(^4F)$	11751.7	-2269.9	-439.0	-601.7	-523.6	-474.6	-545.8
	$^2P(^4F)$	15222.9	-3540.2	-1298.8	-653.4	-495.9	-307.8	-463.0
	$^2H(^4F)$	16564.6	-2085.4	8.7	-680.2	-606.2	-610.5	-652.7
	$^2D(^4F)$	16008.0	-4169.5	-1548.1	-652.3	-403.9	-156.1	-352.5
Cr(II)	$^3H(^5D)$	17064.7	-1793.5	77.7	-917.2	-881.8	-890.7	-838.6
	$^3P(^5D)$	17152.3	-3777.4	-1748.3	-1023.4	-790.3	-513.4	-586.4
	$^3F(^5D)$	18176.7	-3483.1	-1130.7	-1002.7	-846.1	-681.4	-708.9
	$^3G(^5D)$	20521.2	-3345.9	-424.9	-802.5	-719.0	-668.6	-696.6
Mn(II)	$^4G(^6S)$	26845.3	-3938.6	-486.9	-1157.6	-1028.9	-974.1	-990.9
	$^4P(^6S)$	29193.2	-6034.9	-2460.2	-1635.0	-1236.6	-863.9	-1002.2
	$^4D(^6S)$	32351.9	-5975.2	-1134.9	-1072.4	-897.8	-755.3	-865.4
Fe(II)	$^3H(^5D)$	19828.9	-1919.8	30.8	-954.5	-922.1	-922.3	-868.7
	$^3P(^5D)$	19609.8	-4592.6	-1839.8	-1014.7	-745.2	-486.1	-571.1
	$^3F(^5D)$	21212.4	-3701.4	-1144.4	-1027.6	-862.5	-709.5	-743.2
	$^3G(^5D)$	24414.4	-3071.6	-266.4	-815.9	-743.7	-685.3	-684.3
Co(II)	$^4P(^4F)$	14561.4	-3073.8	-631.5	-156.4	-95.5	-35.5	-171.9
	$^2G(^4F)$	16512.2	-2046.0	-377.2	-639.1	-548.2	-500.3	-493.2
	$^2P(^4F)$	19620.5	-4982.9	-1243.6	-517.3	-327.3	-179.9	-306.7
	$^2H(^4F)$	22228.0	-2155.6	104.5	-628.4	-565.8	-580.3	-587.0
	$^2D(^4F)$	22713.9	-3815.3	-1267.0	-623.6	-375.9	-149.6	-233.8
Ni(II)	$^1D(^3F)$	13037.6	-3078.9	-1020.0	-736.9	-497.0	-378.2	-519.8
	$^3P(^3F)$	15836.3	-2893.9	-216.5	64.8	225.2	277.8	68.6
	$^1G(^3F)$	22114.8	-2649.3	-98.9	-621.5	-365.2	-356.1	-571.0

Table S4: Difference excitation energies in cm^{-1} with respect to experimental values obtained from NIST for 3d (III) metal ion relative to the respective ground state at various multireference methodologies utilizing 3d and 4d orbitals in the active space in a basis of DKH-def2-QZVPP.

Ion	State	Exp.	CASSCF	NEVPT2	NEVPT3	NEVPT4	MRCEPA(0)	MRCCSD
V(III)	$^1D(^3F)$	10536.0	-2617.3	-922.0	-412.1	-243.8	-149.2	-355.7
	$^3P(^3F)$	12924.6	-2952.1	-847.0	-0.7	146.7	205.3	58.3
	$^1G(^3F)$	17967.9	-2517.2	-344.8	-537.9	-382.9	-370.4	-526.2
	$^1S(^3F)$	42038.8	-7619.1	-4043.2	-1786.2	-831.4	-30.6	-696.9
Cr(III)	$^4P(^4F)$	13757.7	-3274.9	-796.0	-38.6	95.3	144.7	-18.3
	$^2G(^4F)$	14699.9	-2438.3	-467.6	-686.6	-607.5	-582.3	-645.3
	$^2P(^4F)$	18919.0	-3932.5	-1482.8	-839.9	-668.2	-513.7	-620.7
	$^2D(^4F)$	20112.2	-4564.5	-1538.7	-624.1	-361.4	-175.8	-350.4
	$^2H(^4F)$	20658.0	-2098.2	45.9	-765.3	-700.5	-732.3	-760.5
Mn(III)	$^3P(^5D)$	21247.7	-3941.6	-1876.8	-1175.0	-914.0	-666.5	-695.6
	$^3H(^5D)$	20959.7	-1685.5	38.8	-1099.1	-1076.3	-1099.5	-1023.9
	$^3F(^5D)$	22344.1	-3670.2	-1260.3	-1190.8	-1019.5	-882.6	-879.1
	$^3G(^5D)$	25152.6	-3457.7	-453.7	-928.9	-843.0	-816.4	-818.5
Fe(III)	$^4G(^1S)$	32281.3	-4042.6	-557.4	-1300.8	-1175.8	-1138.4	-1137.5
	$^4P(^1S)$	35305.8	-6394.0	-2657.9	-1821.4	-1400.7	-1076.8	-1172.9
	$^4D(^1S)$	38869.6	-6305.0	-1207.6	-1175.1	-983.5	-860.2	-943.5
Co(III)	$^3P(^5D)$	23166.7	-5079.5	-1983.9	-1109.2	-817.0	-579.7	-674.6
	$^3H(^5D)$	23341.5	-2004.4	-71.3	-1112.2	-1082.2	-1097.0	-1044.6
	$^3F(^5D)$	25028.7	-4032.3	-1298.7	-1174.8	-995.6	-857.5	-893.8
	$^3G(^5D)$	28814.2	-3189.4	-298.1	-875.4	-791.6	-739.5	-742.9
Ni(III)	$^4P(^4F)$	17189.3	-3240.0	-682.6	-197.2	-123.0	-68.3	-173.4
	$^2G(^4F)$	19174.5	-2209.3	-484.5	-741.8	-644.3	-597.3	-591.9
	$^2P(^4F)$	22831.0	-5508.8	-1328.0	-544.9	-333.6	-193.6	-324.5
	$^2H(^4F)$	25964.6	-2178.5	81.2	-679.7	-618.3	-640.1	-633.1
	$^2D(^4F)$	26616.9	-4017.8	-1288.0	-595.5	-322.5	-106.1	-186.5
Cu(III)	$^1D(^3F)$	14894.8	-3402.9	-1245.3	-875.7	-681.4	-571.6	-652.4
	$^3P(^3F)$	18557.4	-2877.9	-74.9	297.0	378.3	414.4	328.0
	$^1G(^3F)$	25559.8	-2778.3	-153.1	-548.3	-440.2	-449.3	-464.8

2 Diatomic Dissociation Potential Energy Surfaces

2.1 MRCCSD comparison

Table S5: Difference energy in kcal/mol in comparison to MRCCSD for NEVPT methods and MRCEPA(0) for the BH molecule in the def2-QZVP basis set.

R(Å)	NEVPT2	NEVPT3	NEVPT4	MRCEPA(0)
0.80	9.91	3.42	1.11	-0.65
0.85	9.88	3.44	1.10	-0.69
0.90	9.79	3.45	1.12	-0.68
0.95	9.68	3.41	1.11	-0.65
1.00	9.54	3.37	1.10	-0.63
1.05	9.39	3.32	1.08	-0.62
1.10	9.24	3.27	1.06	-0.62
1.15	9.10	3.22	1.04	-0.61
1.20	8.96	3.17	1.02	-0.61
1.25	8.82	3.12	0.99	-0.61
1.30	8.68	3.06	0.97	-0.62
1.35	8.54	3.01	0.94	-0.61
1.40	8.41	2.96	0.92	-0.61
1.45	8.29	2.91	0.90	-0.61
1.50	8.16	2.86	0.87	-0.61
1.55	8.04	2.81	0.85	-0.62
1.60	7.92	2.77	0.82	-0.62
1.70	7.70	2.67	0.78	-0.62
1.80	7.49	2.59	0.74	-0.62
1.85	7.40	2.55	0.72	-0.62
1.90	7.31	2.51	0.70	-0.63
1.95	7.22	2.48	0.68	-0.63
2.00	7.14	2.44	0.67	-0.63
2.05	7.07	2.41	0.65	-0.63
2.10	6.99	2.38	0.64	-0.63
2.15	6.93	2.35	0.62	-0.63
2.20	6.86	2.32	0.61	-0.64
2.30	6.74	2.27	0.58	-0.64
2.40	6.63	2.21	0.56	-0.64
2.50	6.53	2.16	0.54	-0.65
2.60	6.44	2.11	0.51	-0.65
2.70	6.38	2.09	0.52	-0.63
2.80	6.31	2.04	0.50	-0.63
2.90	6.24	2.00	0.48	-0.64
3.00	6.18	1.95	0.45	-0.64
3.25	6.05	1.86	0.41	-0.65
3.50	5.96	1.79	0.37	-0.65
3.75	5.89	1.74	0.35	-0.65
4.00	5.85	1.71	0.33	-0.65
4.50	5.82	1.68	0.31	-0.66
5.00	5.81	1.68	0.31	-0.66
5.50	5.75	1.62	0.25	-0.71
6.00	5.75	1.62	0.25	-0.71
0 Max	9.91	3.45	1.12	-0.61
Min	5.75	1.62	0.25	-0.71
MAE	7.51	2.53	0.71	-0.64
σ	1.34	0.59	0.27	0.02

Table S6: Difference energy in kcal/mol in comparison to MRCCSD for NEVPT methods and MRCEPA(0) for the CO molecule in the def2-QZVP basis set.

**R(Å)**	**NEVPT2**	**NEVPT3**	**NEVPT4**	**MRCEPA(0)**
0.80	20.87	5.18	-0.51	-2.72
0.85	20.76	5.06	-0.63	-2.85
0.90	20.69	4.98	-0.73	-2.97
0.95	20.73	5.02	-0.72	-2.98
1.00	20.76	5.06	-0.71	-2.99
1.05	20.76	5.09	-0.70	-3.01
1.10	20.75	5.12	-0.70	-3.03
1.15	20.69	5.12	-0.72	-3.06
1.20	20.62	5.11	-0.75	-3.09
1.25	20.54	5.11	-0.76	-3.10
1.30	20.45	5.10	-0.78	-3.11
1.35	20.34	5.06	-0.82	-3.13
1.40	20.21	5.02	-0.87	-3.16
1.45	20.09	4.97	-0.92	-3.17
1.50	19.97	4.91	-0.98	-3.20
1.55	19.86	4.85	-1.04	-3.22
1.60	19.77	4.79	-1.11	-3.25
1.70	19.70	4.66	-1.24	-3.30
1.80	19.82	4.54	-1.35	-3.36
1.85	19.98	4.50	-1.38	-3.39
1.90	20.19	4.47	-1.41	-3.41
1.95	20.47	4.44	-1.42	-3.44
2.00	20.79	4.42	-1.42	-3.47
2.05	21.16	4.42	-1.40	-3.49
2.10	21.56	4.42	-1.37	-3.51
2.15	21.97	4.42	-1.34	-3.52
2.20	22.39	4.44	-1.29	-3.54
2.30	23.19	4.47	-1.20	-3.56
2.40	23.88	4.50	-1.11	-3.59
2.50	24.43	4.52	-1.04	-3.61
2.60	24.83	4.53	-0.99	-3.63
2.70	25.10	4.52	-0.96	-3.65
2.80	25.26	4.49	-0.95	-3.68
2.90	24.96	4.53	-0.76	-3.40
3.00	25.00	4.57	-0.70	-3.35
3.25	25.05	4.63	-0.62	-3.28
3.50	24.97	4.55	-0.69	-3.34
3.75	25.01	4.60	-0.63	-3.28
4.00	24.99	4.59	-0.64	-3.29
4.50	24.73	4.34	-0.89	-3.53
5.00	25.24	4.89	-0.33	-2.95
5.50	25.24	5.06	-0.09	-2.65
6.00	25.34	5.17	0.02	-2.54
Max	25.34	5.18	0.02	-2.54
Min	19.70	4.34	-1.42	-3.68
MAE	22.17	4.75	-0.90	-3.25
σ	2.16	0.28	0.35	0.28

Table S7: Difference energy in kcal/mol in comparison to MRCCSD for NEVPT methods and MRCEPA(0) for the F_2 molecule in the def2-QZVP basis set.

**R(Å)**	**NEVPT2**	**NEVPT3**	**NEVPT4**	**MRCEPA(0)**
0.85	28.55	5.75	-1.62	-3.73
0.90	28.71	6.06	-1.73	-3.90
0.95	28.31	6.16	-1.87	-4.00
1.00	27.81	6.21	-2.01	-4.08
1.05	27.44	6.28	-2.11	-4.11
1.10	27.20	6.31	-2.23	-4.18
1.15	27.16	6.34	-2.34	-4.25
1.20	27.33	6.36	-2.42	-4.32
1.25	27.71	6.38	-2.46	-4.37
1.30	28.25	6.40	-2.46	-4.41
1.35	28.94	6.40	-2.44	-4.44
1.40	29.73	6.40	-2.38	-4.46
1.45	30.59	6.39	-2.30	-4.46
1.50	31.49	6.38	-2.20	-4.46
1.55	32.39	6.36	-2.09	-4.44
1.60	33.26	6.33	-1.98	-4.43
1.70	34.84	6.28	-1.76	-4.40
1.80	36.09	6.19	-1.61	-4.41
1.85	36.63	6.19	-1.52	-4.38
1.90	37.08	6.16	-1.46	-4.39
1.95	37.45	6.14	-1.41	-4.39
2.00	37.76	6.12	-1.37	-4.39
2.05	38.01	6.10	-1.33	-4.40
2.10	38.22	6.09	-1.30	-4.40
2.15	38.39	6.08	-1.28	-4.40
2.20	38.52	6.07	-1.26	-4.40
2.30	38.71	6.06	-1.23	-4.41
2.40	38.82	6.05	-1.21	-4.41
2.50	38.89	6.04	-1.20	-4.40
2.60	38.91	6.03	-1.19	-4.40
2.70	38.89	6.02	-1.18	-4.39
2.80	38.80	5.98	-1.18	-4.37
2.90	38.81	5.98	-1.18	-4.37
3.00	38.81	5.98	-1.18	-4.37
3.25	38.81	5.98	-1.18	-4.36
3.50	38.81	5.98	-1.18	-4.36
3.75	38.80	5.98	-1.18	-4.36
4.00	38.79	5.97	-1.18	-4.36
4.50	38.79	5.97	-1.18	-4.37
5.00	38.78	5.96	-1.19	-4.38
5.50	38.76	5.94	-1.21	-4.40
6.00	38.71	5.89	-1.26	-4.44
Max	38.91	6.40	-1.18	-3.01
Min	22.34	3.95	-2.46	-4.46
MAE	34.44	6.09	-1.62	-4.31
σ	5.04	0.37	0.47	0.25

Table S8: Difference energy in kcal/mol in comparison to MRCCSD for NEVPT methods and MRCEPA(0) for the HF molecule in the def2-QZVP basis set.

**R(Å)**	**NEVPT2**	**NEVPT3**	**NEVPT4**	**MRCEPA(0)**
0.80	14.36	4.73	-1.19	-2.30
0.85	14.06	4.80	-1.24	-2.35
0.90	13.76	4.84	-1.29	-2.40
0.95	13.50	4.85	-1.34	-2.43
1.00	13.30	4.85	-1.39	-2.46
1.05	13.19	4.83	-1.44	-2.49
1.10	13.15	4.81	-1.47	-2.52
1.15	13.18	4.78	-1.50	-2.54
1.20	13.28	4.74	-1.52	-2.56
1.25	13.44	4.69	-1.53	-2.57
1.30	13.66	4.64	-1.54	-2.58
1.35	13.93	4.58	-1.53	-2.58
1.40	14.24	4.52	-1.51	-2.58
1.45	14.58	4.45	-1.48	-2.57
1.50	14.94	4.38	-1.44	-2.55
1.55	15.32	4.31	-1.39	-2.53
1.60	15.70	4.23	-1.34	-2.50
1.70	16.45	4.06	-1.21	-2.44
1.80	17.14	3.89	-1.08	-2.37
1.85	17.45	3.82	-1.02	-2.34
1.90	17.74	3.74	-0.96	-2.31
1.95	18.00	3.67	-0.90	-2.28
2.00	18.23	3.61	-0.84	-2.26
2.05	18.44	3.55	-0.80	-2.24
2.10	18.62	3.49	-0.75	-2.22
2.15	18.78	3.44	-0.71	-2.20
2.20	18.92	3.40	-0.68	-2.18
2.30	19.14	3.33	-0.62	-2.16
2.40	19.30	3.28	-0.58	-2.14
2.50	19.41	3.24	-0.54	-2.13
2.60	19.49	3.22	-0.52	-2.12
2.70	19.55	3.20	-0.50	-2.11
2.80	19.58	3.18	-0.49	-2.10
2.90	19.61	3.17	-0.49	-2.10
3.00	19.62	3.17	-0.48	-2.10
3.25	19.64	3.16	-0.47	-2.10
3.50	19.65	3.15	-0.47	-2.09
3.75	19.65	3.15	-0.47	-2.09
4.00	19.57	3.07	-0.55	-2.17
4.50	19.54	3.04	-0.57	-2.18
5.00	19.54	3.04	-0.57	-2.18
5.50	19.54	3.04	-0.57	-2.18
6.00	19.54	3.04	-0.57	-2.18
Max	19.65	4.85	-0.47	-2.05
Min	13.15	3.04	-1.54	-2.58
MAE	16.85	3.88	-0.97	-2.31
σ	2.60	0.68	0.40	0.18

Table S9: Difference energy in kcal/mol in comparison to MRCCSD for NEVPT methods and MRCEPA(0) for the N_2 molecule in the def2-QZVP basis set.

**R(Å)**	**NEVPT2**	**NEVPT3**	**NEVPT4**	**MRCEPA(0)**
0.80	20.22	4.55	-0.71	-2.57
0.85	20.09	4.39	-0.86	-2.70
0.90	20.05	4.33	-0.90	-2.74
0.95	20.01	4.29	-0.94	-2.78
1.00	19.97	4.26	-0.97	-2.80
1.05	19.92	4.23	-1.01	-2.84
1.10	19.85	4.20	-1.04	-2.87
1.15	19.78	4.16	-1.09	-2.92
1.20	19.70	4.12	-1.13	-2.96
1.25	19.63	4.09	-1.17	-3.00
1.30	19.56	4.05	-1.22	-3.04
1.35	19.51	4.02	-1.25	-3.07
1.40	19.46	3.99	-1.30	-3.12
1.45	19.42	3.95	-1.35	-3.16
1.50	19.40	3.90	-1.40	-3.21
1.55	19.41	3.84	-1.46	-3.27
1.60	19.44	3.77	-1.52	-3.33
1.70	19.57	3.58	-1.67	-3.48
1.80	19.85	3.37	-1.78	-3.60
1.85	20.03	3.28	-1.81	-3.64
1.90	20.21	3.19	-1.84	-3.68
1.95	20.38	3.11	-1.86	-3.71
2.00	20.55	3.04	-1.87	-3.73
2.05	20.72	2.99	-1.86	-3.74
2.10	20.87	2.95	-1.85	-3.74
2.15	21.02	2.93	-1.83	-3.73
2.20	21.15	2.91	-1.81	-3.72
2.30	21.36	2.90	-1.78	-3.70
2.40	21.53	2.89	-1.75	-3.69
2.50	21.64	2.88	-1.74	-3.69
2.60	21.72	2.87	-1.74	-3.69
2.70	21.77	2.85	-1.75	-3.71
2.80	21.79	2.82	-1.76	-3.72
2.90	21.82	2.82	-1.77	-3.73
3.00	21.82	2.80	-1.78	-3.74
3.25	21.82	2.77	-1.80	-3.76
3.50	21.83	2.77	-1.79	-3.75
3.75	21.83	2.77	-1.79	-3.74
4.00	21.86	2.80	-1.76	-3.72
4.50	21.85	2.78	-1.77	-3.72
5.00	21.86	2.80	-1.75	-3.71
5.50	21.48	2.41	-2.14	-4.09
6.00	21.46	2.40	-2.16	-4.11
Max	21.86	4.55	-0.71	-2.57
Min	19.40	2.40	-2.16	-4.11
MAE	20.63	3.39	-1.55	-3.43
σ	0.94	0.64	0.37	0.41

Table S10: Difference energy in kcal/mol in comparison to MRCCSD for NEVPT methods and MRCEPA(0) for the O_2 molecule in the def2-QZVP basis set.

**R(Å)**	**NEVPT2**	**NEVPT3**	**NEVPT4**	**MRCEPA(0)**
0.80	22.12	4.29	-2.04	-3.83
0.85	22.33	4.30	-2.21	-4.02
0.90	22.58	4.38	-2.31	-4.15
0.95	22.83	4.47	-2.40	-4.28
1.00	22.89	4.38	-2.66	-4.57
1.05	23.17	4.50	-2.68	-4.64
1.10	23.49	4.65	-2.65	-4.66
1.15	23.78	4.76	-2.65	-4.71
1.20	24.08	4.85	-2.64	-4.75
1.25	24.38	4.93	-2.63	-4.79
1.30	24.56	4.86	-2.75	-4.96
1.35	25.03	5.05	-2.59	-4.85
1.40	25.43	5.13	-2.54	-4.85
1.45	25.84	5.19	-2.50	-4.85
1.50	26.29	5.25	-2.44	-4.84
1.55	26.79	5.30	-2.38	-4.83
1.60	27.34	5.35	-2.30	-4.81
1.70	28.58	5.44	-2.09	-4.73
1.80	29.92	5.55	-1.82	-4.62
1.85	30.59	5.61	-1.67	-4.56
1.90	31.23	5.67	-1.52	-4.49
1.95	31.82	5.74	-1.37	-4.43
2.00	32.33	5.80	-1.24	-4.38
2.05	32.76	5.85	-1.13	-4.33
2.10	33.10	5.89	-1.04	-4.30
2.15	33.37	5.93	-0.96	-4.27
2.20	33.57	5.95	-0.91	-4.25
2.30	33.83	5.96	-0.86	-4.24
2.40	34.24	6.22	-0.57	-3.97
2.50	34.07	5.96	-0.81	-4.22
2.60	34.11	5.95	-0.82	-4.23
2.70	34.12	5.93	-0.83	-4.24
2.80	34.12	5.91	-0.84	-4.25
2.90	34.18	5.96	-0.78	-4.20
3.00	34.17	5.94	-0.79	-4.20
3.25	33.97	5.74	-0.98	-4.39
3.50	33.80	5.58	-1.14	-4.54
3.75	33.78	5.56	-1.16	-4.56
4.00	33.76	5.54	-1.18	-4.57
4.50	33.60	5.38	-1.33	-4.72
5.00	33.55	5.33	-1.38	-4.77
5.50	33.45	5.23	-1.48	-4.87
6.00	33.45	5.23	-1.48	-4.87
Max	34.24	6.22	-0.57	-3.83
Min	22.12	4.29	-2.75	-4.96
MAE	29.59	5.36	-1.69	-4.50
σ	4.59	0.55	0.73	0.29

2.2 FCI comparison

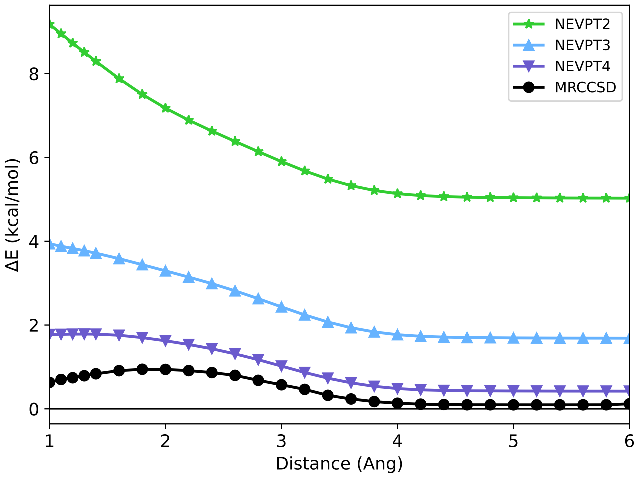

(a) BH CAS(5,4) (aug-cc-pVQZ)

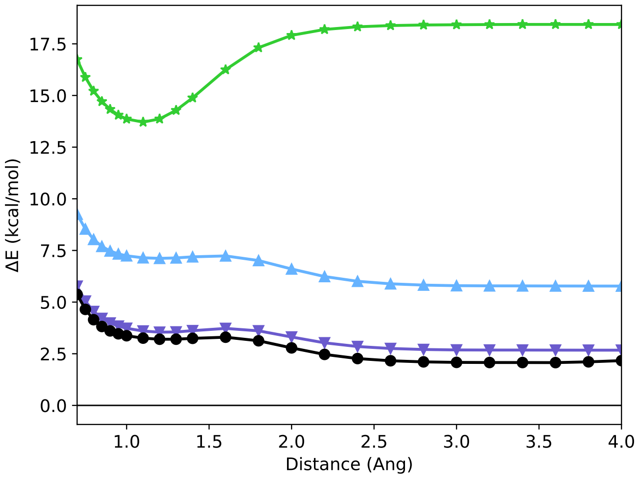

(b) HF CAS(5,8) (6-31G**)

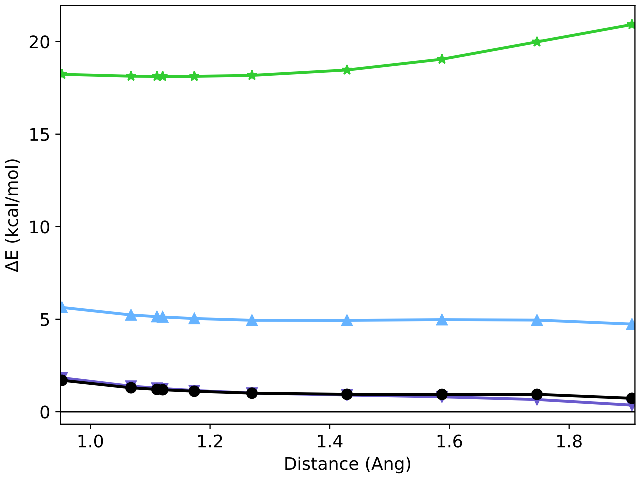

(c) N_2 CAS(8,10) (cc-pVDZ)

Figure S1: Non-parallelity errors along diatomic bond dissociation curves relative to FCI with active space sizes and basis sets shown in parenthesis.

Table S11: Difference energy values (reported in kcal/mol) between FCI and NEVPT2, NEVPT3, NEVPT4, and MRCCSD inlcuding maximum, minimum, mean average error (reported in kcal/mol) and standard deviation for the BH molecule using the aug-cc-pVQZ basis set. FCI results were taken from Dutta, A.; Sherrill, C. D. *J. Chem. Phys.*,**2003**,118, 1610-1619.

**R(Å)**	**NEVPT2**	**NEVPT3**	**NEVPT4**	**MRCCSD**
1.00	9.18	3.94	1.78	0.63
1.10	8.95	3.88	1.78	0.70
1.20	8.73	3.83	1.78	0.74
1.30	8.51	3.77	1.78	0.79
1.40	8.29	3.72	1.78	0.84
1.60	7.88	3.58	1.76	0.91
1.80	7.50	3.44	1.70	0.94
2.00	7.18	3.29	1.62	0.94
2.20	6.89	3.14	1.54	0.91
2.40	6.63	2.99	1.43	0.87
2.60	6.38	2.82	1.31	0.80
2.80	6.14	2.63	1.17	0.68
3.00	5.90	2.43	1.02	0.57
3.20	5.68	2.24	0.87	0.46
3.40	5.48	2.07	0.73	0.32
3.60	5.33	1.94	0.62	0.24
3.80	5.21	1.84	0.54	0.17
4.00	5.14	1.77	0.48	0.13
4.20	5.09	1.73	0.45	0.11
4.40	5.07	1.71	0.44	0.10
4.60	5.05	1.70	0.43	0.10
4.80	5.04	1.70	0.43	0.10
5.00	5.04	1.69	0.43	0.10
5.20	5.04	1.69	0.43	0.10
5.40	5.03	1.69	0.42	0.10
5.60	5.03	1.69	0.42	0.10
5.80	5.03	1.69	0.42	0.10
6.00	5.03	1.69	0.42	0.12
Max.	9.18	3.94	1.78	0.94
Min.	5.03	1.69	0.42	0.10
MAE	6.27	2.51	1.00	0.45
σ	1.4364	0.8668	0.5795	0.3432

Table S12: Difference energy values (reported in kcal/mol) between FCI and NEVPT2, NEVPT3, NEVPT4, and MRCCSD inlcuding maximum, minimum, mean average error (reported in kcal/mol) and standard deviation for the N_2 molecule using the cc-pVDZ basis set. FCI results were taken from Hanauer, M.; Köhn, A. *J. Chem. Phys.*,**2011**,134.

**R(Å)**	**NEVPT2**	**NEVPT3**	**NEVPT4**	**MRCCSD**
0.95	18.23	5.64	1.83	1.70
1.07	18.13	5.23	1.39	1.30
1.11	18.12	5.14	1.28	1.21
1.12	18.12	5.12	1.26	1.19
1.17	18.12	5.04	1.15	1.11
1.27	18.17	4.94	1.01	1.00
1.43	18.47	4.94	0.90	0.94
1.59	19.05	4.97	0.81	0.94
1.75	19.99	4.95	0.66	0.94
1.90	20.92	4.74	0.36	0.73
Max.	20.92	5.64	1.83	1.70
Min.	18.12	4.74	0.36	0.73
MAE	18.73	5.07	1.06	1.11
σ	0.9759	0.2407	0.4135	0.2669

Table S13: Difference energy values (reported in kcal/mol) between FCI and NEVPT2, NEVPT3, NEVPT4, and MRCCSD inlcuding maximum, minimum, mean average error (reported in kcal/mol) and standard deviation for the HF molecule using the 6-31G** basis set. FCI results were taken from Dutta, A.; Sherrill, C. D. *J. Chem. Phys.*,**2003**,118, 1610-1619.

**R(Å)**	**NEVPT2**	**NEVPT3**	**NEVPT4**	**MRCCSD**
0.70	16.74	9.25	5.77	5.37
0.75	15.88	8.54	5.05	4.65
0.80	15.21	8.03	4.55	4.15
0.85	14.71	7.70	4.21	3.82
0.90	14.33	7.47	3.99	3.61
0.95	14.05	7.33	3.84	3.47
1.00	13.86	7.24	3.73	3.37
1.10	13.71	7.14	3.60	3.26
1.20	13.87	7.12	3.54	3.20
1.30	14.28	7.14	3.56	3.20
1.40	14.89	7.19	3.62	3.25
1.60	16.25	7.24	3.73	3.30
1.80	17.31	7.01	3.61	3.13
2.00	17.91	6.60	3.31	2.78
2.20	18.19	6.24	3.03	2.47
2.40	18.32	6.01	2.85	2.27
2.60	18.38	5.89	2.76	2.16
2.80	18.41	5.82	2.71	2.11
3.00	18.42	5.79	2.68	2.08
3.20	18.43	5.79	2.68	2.08
3.40	18.44	5.78	2.68	2.07
3.60	18.44	5.78	2.68	2.07
3.80	18.44	5.78	2.67	2.11
4.00	18.44	5.78	2.67	2.17
Max.	18.44	9.25	5.77	5.37
Min.	13.71	5.78	2.67	2.07
MAE	16.54	6.82	3.48	3.01
σ	1.9083	0.9777	0.8221	0.9002

3 Ethylene Double Bond Rotation

Table S14: MRCCSD energy values in Hartree for the rotation of the ethylene double bond with a basis of def2-TZVP.

HCCH Angle	MRCCSD
0	-78.41919072
10	-78.41725731
20	-78.41147190
30	-78.40189375
40	-78.38865520
50	-78.37201490
60	-78.35248598
70	-78.33122368
80	-78.31145727
90	-78.30210088
100	-78.31145727
110	-78.33122368
120	-78.35248598
130	-78.37201490
140	-78.38865520
150	-78.40189375
160	-78.41147190
170	-78.41725731
180	-78.41919072

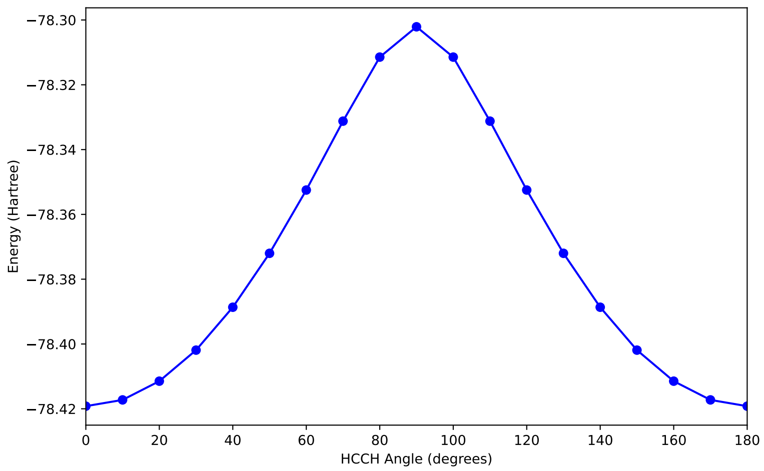

Figure S2: Graphed MRCCSD energy values in Hartree for the rotation of ethylene along the double bond at a basis of def2-TZVP.

Table S15: Difference energy values in kcal/mol in comparison to MRCCSD for NEVPT method for the rotation of the ethylene double bond with a basis of def2-TZVP.

**HCCH Angle**	**NEVPT2**	**NEVPT3**	**NEVPT4**	**MRCEPA(0)**
0	17.20	2.27	-3.72	-5.85
10	17.23	2.28	-3.72	-5.87
20	17.31	2.33	-3.71	-5.91
30	17.44	2.40	-3.69	-5.97
40	17.64	2.49	-3.67	-6.07
50	17.92	2.58	-3.65	-6.22
60	18.30	2.65	-3.63	-6.41
70	18.82	2.63	-3.64	-6.67
80	19.47	2.41	-3.71	-6.95
90	19.85	2.19	-3.77	-7.07
100	19.47	2.41	-3.71	-6.95
110	18.82	2.63	-3.64	-6.67
120	18.30	2.65	-3.63	-6.41
130	17.92	2.58	-3.65	-6.22
140	17.64	2.49	-3.67	-6.07
150	17.44	2.40	-3.69	-5.97
160	17.31	2.33	-3.71	-5.91
170	17.23	2.28	-3.72	-5.87
180	17.20	2.27	-3.72	-5.85
MAE	18.03	2.43	-3.69	-6.26
σ	0.87	0.15	0.04	0.42

3.1 Example NEVPT4 ORCA Calculation

```
!def2-TZVP NoPop fic-nevpt4(sd)
%casscf nel         2
        norb        2
        mult        1
        nroots      1
end
%paras
cc2 = 1.339
hc3 = 1.087
dih4 = 180.000
hcc5 = 121.300
dih5 = 180.000
dih6 = 0.000 rot = 0,180,19
end
*int 0 1
c 0 0 0 0 0 0
c 1 0 0 {cc2} 0 0
h 1 2 0 {hc3} {hcc5} 0
h 1 2 3 {hc3} {hcc5} {dih4}
h 2 1 3 {hc3} {hcc5} {dih5+rot} h 2 1 3 {hc3} {hcc5} {dih6+rot}
*
```

Listing S1: Example of ethylene double bond twisting with the NEVPT4 methodology.

3.2 Example FIC-MRCCSD ORCA Calculation

```
!def2-TZVP NoPop fic-mrcc
%casscf nel         2
        norb        2
        mult        1
        nroots      1
end
%paras
cc2 = 1.339
hc3 = 1.087
dih4 = 180.000
hcc5 = 121.300
dih5 = 180.000
dih6 = 0.000 rot = 0,180,19
end
*int 0 1
c 0 0 0 0 0 0
c 1 0 0 {cc2} 0 0
h 1 2 0 {hc3} {hcc5} 0
h 1 2 3 {hc3} {hcc5} {dih4}
h 2 1 3 {hc3} {hcc5} {dih5+rot} h 2 1 3 {hc3} {hcc5} {dih6+rot}
*
```

Listing S2: Example of ethylene double bond twisting with the FIC-MRCCSD methodology.

4 Size Consistency

Firstly, it is important to specify the active space sizes used for each of the five dimer systems studied. The ethylene dimer utilized an active space of (4, 4), with 2 electrons and 2 orbitals allocated for each monomer. For the butadiene dimer, we employed an (8, 8) active space, where each monomer was represented by 4 electrons and 4 orbitals. In the case of hexatriene, both monomers were described using 6 electrons and 6 orbitals, resulting in a (12, 12) active space for the dimer. Notably, the active space sizes for the monomers remained consistent, regardless of the differences in molecular size. Consequently, the overall active spaces for the combined systems were (6, 6) for ethylene and butadiene, and (8, 8) for ethylene and hexatriene. Calculations here utilize the AVAS methodology within the ORCA 6.0 framework.([Sayfutyarova, E. R.; Hammes-Schiffer, S. *J. Chem. Theory Comput.*,**2019**,*15*,1679–1689.], [Sayfutyarova, E. R.; Sun, Q.; Chan, G. K.-L.; Knizia, G. *J. Chem. Theory Comput.*,**2017**,*13*,4063–4078.])

Table S16: Monomer and dimer absolute energies in hartree with a basis set of def2-SVP.

**Monomer**		**CASSCF**	**NEVPT2**	**NEVPT3**	**NEVPT4**
Ethene		-78.005875	-78.256792	-78.280562	-78.288816
Butadiene		-156.011750	-156.513585	-156.561125	-156.577632
Hexatriene		-231.724284	-232.446226	-232.498077	-232.521308
**Dimer**		**CASSCF**	**NEVPT2**	**NEVPT3**	**NEVPT4**
Ethene	Ethene	-156.011750	-156.513585	-156.561125	-156.577632
Butadiene	Butadiene	-309.729540	-310.701238	-310.777303	-310.808716
Hexatriene	Hexatriene	-463.448568	-464.892451	-464.996153	-465.042617
Butadiene	Ethene	-232.870645	-233.607411	-233.669214	-233.693174
Hexatriene	Ethene	-309.730159	-310.703018	-310.778639	-310.810125

Table S17: Cartesian coordinates for ethylene dimer.

	x (Å)	y (Å)	z (Å)
C	-9.271	-0.808	0.000
C	-8.060	-0.270	0.000
H	-9.594	-1.484	0.784
H	-9.989	-0.597	-0.784
H	-7.342	-0.481	0.784
H	-7.737	0.405	-0.784
C	-9.271	-0.808	100.000
C	-8.060	-0.270	100.000
H	-9.594	-1.484	100.784
H	-9.989	-0.597	99.216
H	-7.342	-0.481	100.784
H	-7.737	0.405	99.216

Table S18: Cartesian coordinates for butadiene dimer.

	x (Å)	y (Å)	z (Å)
C	-9.296	-0.811	-0.116
C	-8.151	-0.130	-0.040
H	-9.539	-1.587	0.601
H	-10.024	-0.615	-0.892
C	-7.138	-0.347	0.978
H	-7.938	0.642	-0.774
C	-5.993	0.334	1.054
H	-7.351	-1.119	1.712
H	-5.750	1.110	0.337
H	-5.266	0.138	1.830
C	-9.296	-0.811	99.884
C	-8.151	-0.130	99.960
H	-9.539	-1.587	100.601
H	-10.024	-0.615	99.108
C	-7.138	-0.347	100.978
H	-7.938	0.642	99.226
C	-5.993	0.334	101.054
H	-7.351	-1.119	101.712
H	-5.750	1.110	100.337
H	-5.266	0.138	101.830

Table S19: Cartesian coordinates for hexatriene dimer.

	x (Å)	y (Å)	z (Å)
C	-9.223	-0.852	-0.177
C	-8.095	-0.153	-0.014
H	-9.551	-1.574	0.562
H	-9.850	-0.723	-1.049
C	-7.215	-0.277	1.125
H	-7.799	0.562	-0.776
C	-6.080	0.427	1.285
H	-7.504	-0.991	1.893
C	-5.200	0.302	2.423
H	-5.791	1.140	0.517
C	-4.072	1.001	2.586
H	-5.496	-0.413	3.186
H	-3.744	1.722	1.847
H	-3.445	0.872	3.458
C	-9.223	-0.852	99.823
C	-8.095	-0.153	99.986
H	-9.551	-1.574	100.562
H	-9.850	-0.723	98.951
C	-7.215	-0.277	101.125
H	-7.799	0.562	99.224
C	-6.080	0.427	101.285
H	-7.504	-0.991	101.893
C	-5.200	0.302	102.423
H	-5.791	1.140	100.517
C	-4.072	1.001	102.586
H	-5.496	-0.413	103.186
H	-3.744	1.722	101.847
H	-3.445	0.872	103.458

Table S20: Cartesian coordinates for ethylene+butadiene dimer.

	x (Å)	y (Å)	z (Å)
C	-9.296	-0.811	-0.116
C	-8.151	-0.130	-0.040
H	-9.539	-1.587	0.601
H	-10.024	-0.615	-0.892
C	-7.138	-0.347	0.978
H	-7.938	0.642	-0.774
C	-5.993	0.334	1.054
H	-7.351	-1.119	1.712
H	-5.750	1.110	0.337
H	-5.266	0.138	1.830
C	-9.271	-0.808	100.000
C	-8.060	-0.270	100.000
H	-9.594	-1.484	100.784
H	-9.989	-0.597	99.216
H	-7.342	-0.481	100.784
H	-7.737	0.405	99.216

Table S21: Cartesian coordinates for ethylene+hexatriene dimer.

	x (Å)	y (Å)	z (Å)
C	-9.223	-0.852	-0.177
C	-8.095	-0.153	-0.014
H	-9.551	-1.574	0.562
H	-9.850	-0.723	-1.049
C	-7.215	-0.277	1.125
H	-7.799	0.562	-0.776
C	-6.080	0.427	1.285
H	-7.504	-0.991	1.893
C	-5.200	0.302	2.423
H	-5.791	1.140	0.517
C	-4.072	1.001	2.586
H	-5.496	-0.413	3.186
H	-3.744	1.722	1.847
H	-3.445	0.872	3.458
C	-9.271	-0.808	100.000
C	-8.060	-0.270	100.000
H	-9.594	-1.484	100.784
H	-9.989	-0.597	99.216
H	-7.342	-0.481	100.784
H	-7.737	0.405	99.216

4.1 Example ORCA Calculation

```
!def2-SVP extremescf fic-nevpt4(sd)

%scf
    avas
        system
        center 0,1,7,8
        shell 2,2,2,2
        l 1,1,1,1
        end
    end
end

%casscf
  nel 4
  norb 4
  mult 1
  nroots 1
end

* xyz 0 1
  C   -9.270     -0.808     -0.000
  C   -8.060     -0.269      0.000
  H   -9.594     -1.483      0.783
  H   -9.988     -0.596     -0.783
  H   -7.342     -0.481      0.783
  H   -7.736      0.405     -0.783
  C   -9.270     -0.808     99.999
  C   -8.060     -0.269     100.00
  H   -9.594     -1.483     100.78
  H   -9.988     -0.596     99.216
  H   -7.342     -0.481     100.78
  H   -7.736      0.405     99.216
  *
```

Listing S3: Example Ethylene Dimer NEVPT4 calculation using the AVAS system for active space selection.

5 Efficiency Test- Single Molecule with Ranging Methodologies

5.1 Timings

Table S22: Timing in seconds for SC- and FIC-NEVPT2 in comparison to NEVPT3, NEVPT4, single iteration MRCEPA(0), MRCEPA(0), and MRCCSD with decapentane(10,10) in a basis set of def2-SVP.

**Method**	**Time (s)**
SC-NEVPT	72.3
FIC-NEVPT2	184.6
NEVPT3	506.8
NEVPT4	507.8
MRCEPA(0)(1 iter)	514.9
MRCEPA(0)	2194.0
MRCCSD	517454.9

5.2 AUTOCI Breakdown

Table S23: Breaddown of AUTOCI Timings in seconds for NEVPT4 and a single iteration MRCEPA(0) for decapentane(10,10) in a basis set of def2-SVP.

**NEVPT4**		**MRCEPA(0) (1 iter)**	
Integral Transformation	35.2	Integral Transformation	34.3
RDM	131.0	RDM	131.9
First-Order Amplitudes	18.4	First-Order Amplitudes	20.3
$\langle K\|V\|1\rangle$	321.7	Constant Intermediates	114.8
$\langle K\|H_0\|1\rangle + E_3 + E_4$	1.5	Single Iteration	213.6
TOTAL	507.8	TOTAL	514.9

6 Single Alkene with ranging Active space

6.1 Timings

Table S24: Timings in seconds for NEVPT methodologies in comparison to a single iteration of MRCEPA(0) for the decapentane molecule with ranging active space with a basis set of def2-SVP.

**Active Space**	**NEVPT2**	**NEVPT3**	**NEVPT4**	**MRCEPA(0)(1 iter)**
2,2	95.4	95.4	95.4	100.8
4,4	106.0	106.0	106.0	114.8
6,6	129.7	129.7	129.7	135.3
8,8	187.3	187.3	187.3	201.9
10,10	507.8	507.8	507.8	514.9

6.2 Example ORCA Calculation

```
! def2-SVP fic-cepa(0)

%pal nprocs 1 end

%casscf
  nel 10
  norb 10
  mult 1
  nroots 1
end

%autoci
  density none
  d4tpre 1e-14  // density truncation turned off to numerical zero.
  maxiter 1     // single interation
  stol 1000     // convergence thresh 1000 to force convergence
end

* xyz 0 1
  C   -8.991     -0.867     -0.125
  C   -7.878     -0.152      0.082
  H   -9.362     -1.562      0.617
  H   -9.560     -0.776     -1.041
  C   -7.074     -0.223      1.276
  H   -7.539      0.534     -0.687
  C   -5.951      0.499      1.477
  H   -7.405     -0.907      2.053
  C   -5.145      0.432      2.660
  H   -5.624      1.181      0.697
  C   -4.020      1.156      2.858
  H   -5.471     -0.249      3.441
  C   -3.215      1.090      4.041
  H   -3.694      1.838      2.077
  C   -2.092      1.813      4.242
  H   -3.542      0.407      4.821
  C   -1.288      1.742      5.436
  H   -1.761      2.497      3.465
  C   -0.175      2.456      5.644
  H   -1.627      1.054      6.206
  H    0.195      3.152      4.901
  H    0.393      2.366      6.560
*
```

Listing S4: Calculation of decapentane molecule with CAS(10,10) active space for a single iteration of MRCEPA(0).

7 Increasing Molecular Size

Another test for NEVPT is to examine molecules with a consistent backbone and increasing carbon chain length to demonstrate that the scaling of NEVPT4 remains unchanged with respect to system size. In this study, a series of compounds are investigated, featuring a butadiene backbone with an increasing number of methylene groups (ranging from 2 to 7) attached to the second carbon atom of butadiene, figure S3. The molecules were examined at the basis set of def2-SVP with an active space that was consistently set to be 4 electrons in 4 orbitals, describing the π orbitals within the butadiene backbone.

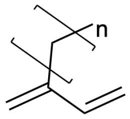

Figure S3: Butadiene backbone with increasing carbon chain length on the second carbon where n ranges from 2 to 7.

The computation times in seconds for the NEVPT4 method are graphically compared to those of FIC-NEVPT2, NEVPT3, and a single iteration of the MRCEPA(0) method, figure S4. As expected, it is observed that computational time increases with carbon chain length. Notably, the NEVPT4 methodology requires about the same time as both NEVPT3 and a single iteration of the MRCEPA(0) method. This finding is significant, as previous sections (1, 2, 3) have established the accuracy of NEVPT4 in relation to the MRCCSD methodology, demonstrating that NEVPT4 can achieve comparable accuracy at a substantially reduced computational cost.

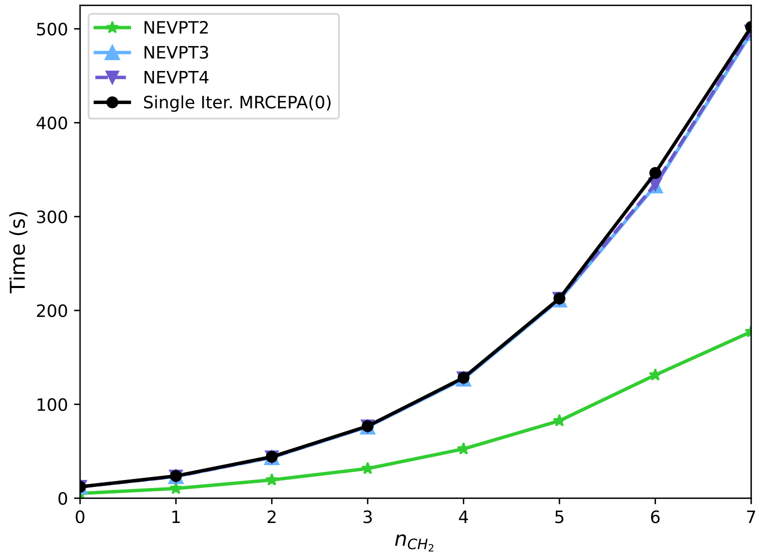

Figure S4: Scaling of NEVPT4 in comparison to NEVPT2, NEVPT3, and a single iteration of MRCEPA(0) for increasing number of methylene groups on a butadiene backbone.

8 Scaling with respect to molecular size

8.1 Timings

Table S25: Timings in seconds for NEVPT methodologies in comparison to a single iteration of MRCEPA(0) for the polyene benchmarking set with a basis set of def2-SVP.

AS	NEVPT2	NEVPT3	NEVPT4	MRCEPA(0)(1 iter)
2,2	0.3	0.9	0.9	0.9
4,4	1.9	5.3	5.4	5.7
6,6	7.1	25.6	25.8	26.6
8,8	22.8	99.0	99.5	99.9
10,10	184.6	506.8	507.8	514.9
12,12	6118.7	7255.2	7257.1	7233.0
14,14	255897.8	259497.2	259500.8	256917.7

Table S26: Timing in seconds of the AUTOCI calculation broken into the NEVPT and RDM parts with the def2-SVP basis set.

Active Space	NEVPT	RDM	TOTAL
2,2	0.6	0.0	1.1
4,4	3.6	0.0	6.5
6,6	16.3	0.1	23.8
8,8	76.7	3.1	99.5
10,10	323.2	129.6	507.8
12,12	1138.4	5938.3	7257.1
14,14	3603	253608.2	259500.8

9 Antiferromagnetically Coupled Copper Complexes

Table S27: Energies in Hartree for singlet and triplet states for the H-He-H molecule using the NEVPT methodologies with the def2-TZVP basis set.

Spin State	CASSCF	NEVPT2	NEVPT3	NEVPT4
Singlet	-3.833600	-3.861914	-3.867361	-3.868582
Triplet	-3.831442	-3.859589	-3.865013	-3.866221

Table S28: Energies in Hartree for singlet and triplet states for $[Cu_2Cl_6]^{-2}$ using the NEVPT methodologies with the def2-TZVP basis set.

Spin State	CASSCF	NEVPT2	NEVPT3	NEVPT4
Singlet	-6035.345435	-6037.647616	-6037.567740	-6037.707210
Triplet	-6035.345488	-6037.647681	-6037.567861	-6037.707342

Table S29: Energies in Hartree for singlet and triplet states for $Cu_2(\mu\text{-}CH_3COO)_4(H_2O)_2$ using the NEVPT methodologies with the def2-TZVP basis set.

Spin State	CASSCF	NEVPT2	NEVPT3	NEVPT4
Singlet	-4339.555379	-4344.377331	-4344.221495	-4344.453438
Triplet	-4339.555293	-4344.377127	-4344.221282	-4344.453152

9.1 Example ORCA Calculation

```
!def2-TZVP fic-nevpt4(sd) noautostart
%casscf nel 2
        norb 2
        mult 3,1
        etol 1e-10
        gtol 1e-4
end
*xyz 0 3
Cu       0.000    0.000    -1.319
Cu       0.000    0.000     1.319
O       -0.090    0.188    -3.458
O       -0.483   -1.925    -1.172
O       -1.865    0.522    -1.063
O       -0.536   -1.901     1.053
O       -1.857    0.548     1.175
O        0.090   -0.188     3.458
O        0.483    1.925     1.172
O        1.865   -0.522     1.063
O        0.536    1.901    -1.053
O        1.857   -0.548    -1.175
C       -0.921   -3.959    -0.042
C       -3.841    1.174     0.060
C       -0.628   -2.488    -0.058
C       -2.412    0.713     0.056
C        0.921    3.959     0.042
C        3.841   -1.174    -0.060
C        0.628    2.488     0.058
C        2.412   -0.713    -0.056
H       -0.026   -0.428    -4.177
H       -1.030   -4.365    -1.034
H       -4.479    0.358    -0.289
H       -0.607    0.930    -3.780
H       -3.950    2.012    -0.634
H       -1.801   -4.171     0.556
H       -4.161    1.426     1.074
H       -0.085   -4.465     0.499
H        0.026    0.428     4.177
H        1.030    4.365     1.034
H        4.479   -0.358     0.289
H        0.607   -0.930     3.780
H        3.950   -2.012     0.634
H        1.801    4.171    -0.556
H        4.161   -1.426    -1.074
H        0.085    4.465    -0.499
*
```

Listing S5: Calculation of copper acetate with the NEVPT4 methodology.
